# Conserved Motifs in the Ligand-Binding Domain of TetR Family Regulators: Identification and Analysis

**DOI:** 10.34133/csbj.0100

**Published:** 2026-05-15

**Authors:** Maria Kepa, Maciej Nielipinski, Estera Widawska, Jakub Filipek, Natalia Kazmierczak, Julia Sosnowska, Krystyna Baca, Maja Nastarowicz, Marcelina Rupp, Mieszko Goslawski-Zeligowski, Julia Mokrzycka, Dominika Nielipinska, Bartosz Sekula, Agnieszka J. Pietrzyk-Brzezinska

**Affiliations:** ^1^ Biotechnology Students Association Ferment, Faculty of Biotechnology and Food Sciences, Lodz University of Technology, Lodz 90-530, Poland.; ^2^ Institute of Molecular and Industrial Biotechnology, Faculty of Biotechnology and Food Sciences, Lodz University of Technology, Lodz 90-537, Poland.

## Abstract

TetR family regulators (TFRs) represent one of the largest and most extensively studied groups of bacterial transcription factors. TFRs contain 2 domains, a conserved N-terminal DNA binding domain and a remarkably variable C-terminal domain (CTD). This structural diversity enables TFRs to recognize a wide range of effectors and regulate numerous processes in bacterial cells. In this study, we identified conserved sequence motifs within the TFR CTDs. We checked these motifs against sequences of the well-characterized TFRs and compiled them into organized data files, which enable direct motif searches in newly characterized TFRs. The detailed motif analysis of 10 TFR representatives revealed that most residues involved in ligand binding exhibit high conservation, with some exceptions in TFRs related to multidrug resistance. Motif mapping onto the CTD structures showed their presence not only in helices α5 to α7 of the central triangle but also in helices α4 or α8 to α9, depending on ligand-binding cavity location. Motifs were also converted into the Prosite patterns for broader usability. Finally, homolog searches across bacterial families indicated a wide distribution of motifs associated with antibiotic and multidrug resistance. These findings provide practical tools for prediction of putative TFR function and may support antimicrobial drug development.

## Graphical Abstract

**Figure:**
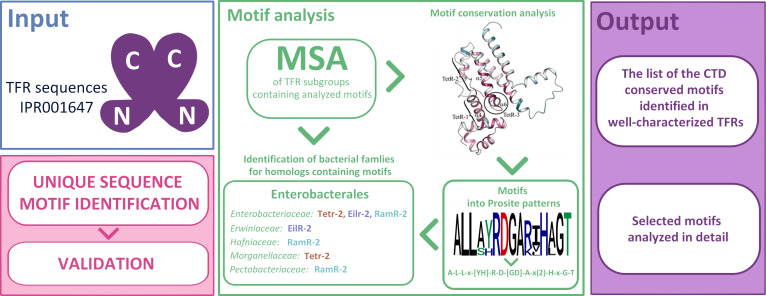

## Introduction

Protein sequence motifs represent unique patterns that may be utilized for the classification of newly discovered proteins into known families. Sequence motifs also enable prediction of protein function using motif databases, while shared motifs across various proteins indicate their functional and structural similarity, which may imply their homology, although in some cases, such similarities may also arise from convergent evolution. In enzymes, sequence motifs are associated with their catalytic function, allowing for the assignment of an enzyme to an appropriate class and a subclass, whereas structural and regulatory proteins contain more divergent motifs [1].

**Fig. 1.**
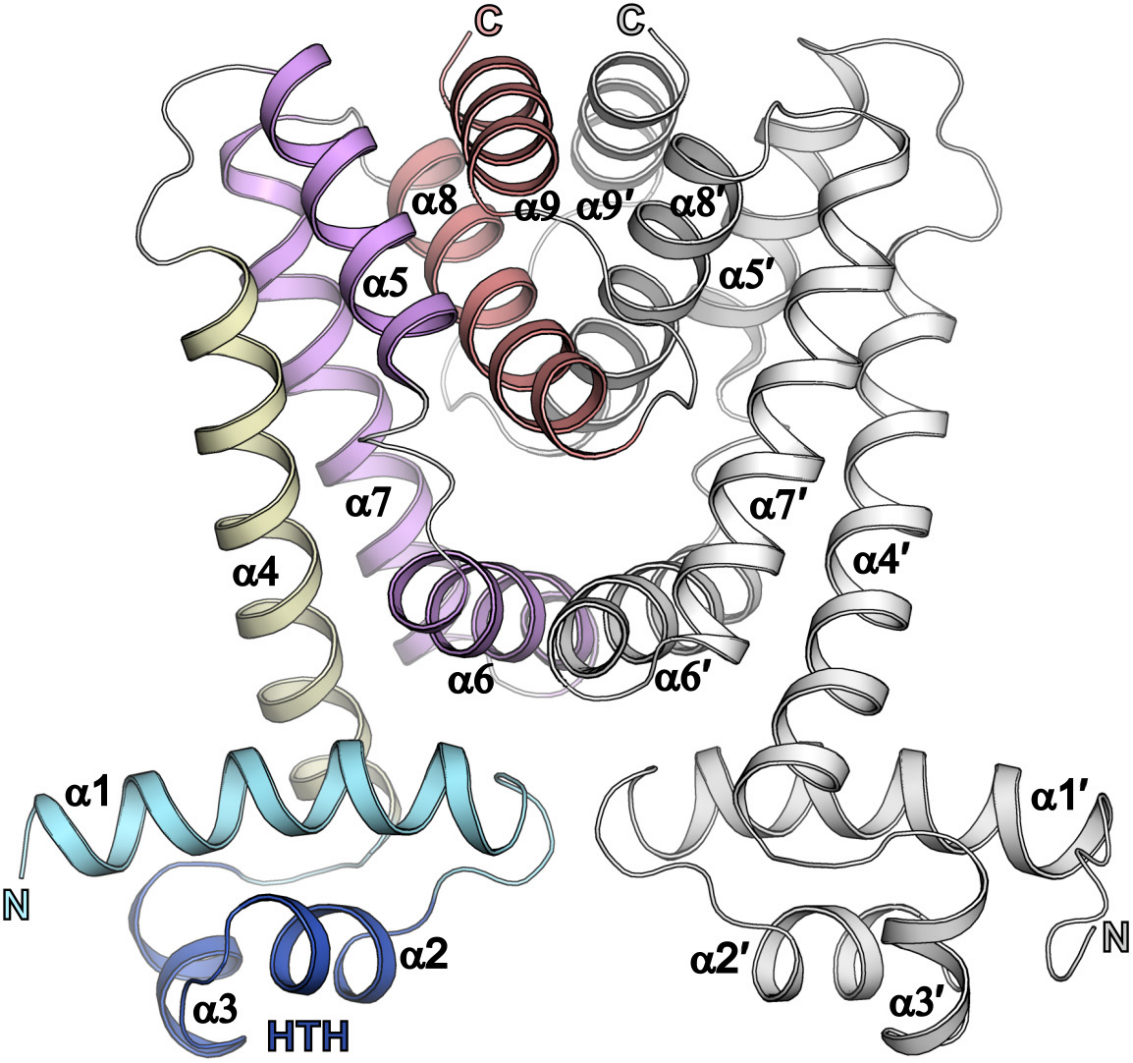
Overall structure of the TetR family regulator (TFR) representative, Eco_RcdA (Protein Data Bank [PDB] ID: 7ACM). One protomer (chain A) of the TFR dimer is presented in colors highlighting the important structural parts of the protein, while the other protomer (chain B) is shown in gray. The N-terminal domain of chain A is shown in blue with the helix-turn-helix (HTH) motif marked in dark blue, and the helices of the C-terminal domain (CTD) are colored according to their functions: helix α4 playing an important role in conformational changes—yellow, helices α5 to α7 forming the central triangle of CTD—purple, and helices α8 and α9 participating in dimerization—red.

Among prokaryotic transcription factors, the most recurrent DNA binding motif is helix-turn-helix (HTH), identified in almost 95% of all known prokaryotic transcription factors [2,3]. The HTH motif is present in all members of the TetR family regulators (TFRs) as a highly conserved 20-amino-acid sequence in the N-terminal DNA binding domain (NTD). This allows for a relatively simple identification of the family members [2,4,5]. In the InterPro database [6], the members of the TetR family are assigned to IPR001647, and this classification is based on the Prosite signature PS01081 covering a conserved region that starts 6 residues upstream the HTH motif and ends 7 residues downstream [7]. The TFR NTD is composed of 3 α-helices, of which helices α2 and α3 form the HTH structural motif (Fig. 1). Helix α2 widens the major groove of the DNA, stabilizes the NTD, and provides the proper orientation of the HTH motif, allowing helix α3 to recognize the target DNA and the formation of base-specific contacts. Helix α1 provides a structural link between the NTD and the ligand-binding domain through conserved interactions with helices α4 and α6 [2,4].

The C-terminal ligand-binding domain (CTD) of TFRs typically consists of helices α4 to α9 (Fig. 1). Helix α4 plays an important role in conformational changes upon DNA or ligand (effector) binding. Helices α5 to α7 create a central triangle and form the ligand-binding cavity in many TFRs. Helices α8 to α9, located at the dimer interface, are crucial for dimerization [2]. In contrast to the NTD, the CTD exhibits substantial sequence and structural diversity. The average pairwise identity among the CTDs of TFRs is only about 9% and, thus, CTD lacks universally conserved motifs across the family [5]. This reflects the adaptation of TFRs to bind a wide variety of ligands, e.g., antibiotics, other antimicrobial compounds, polyphenols, bile acids, cholesterol-coenzyme A derivatives, fatty acids, and many others, based on the TFR origin and biological function. TFRs are involved in the regulation of many different processes in bacteria, like antibiotic and multidrug resistance, control of metabolism in response to changing environmental conditions, or cell-to-cell signaling [2,3,8].

The functional versatility of TFRs has made this family the subject of intensive bioinformatic and structural analyses for many years. Cuthbertson et al. [9] presented the first systematic phylogenetic analysis of TetR-type regulators. The analysis covered 4,243 TFRs encoded in the genomes of 211 prokaryotic species, including model species, as well as pathogenic, commensal, and environmental isolates to maintain high phylogenetic diversity. This approach enabled the identification of a function and ligand for a *Streptomyces coelicolor* TFR of unknown function [9]. Eleven years later, in 2024, our group conducted a much broader phylogenetic analysis, covering the 644,896 unique TFR sequences available in the UniProt database [8]. Based on the calculated phylogenetic tree, we proposed a classification of TFRs into 14 subfamilies, showing their evolutionary relationships and structural differences. Our review revealed substantial differences in the architecture of the TFR ligand pockets located in the CTDs, concerning their size, shape, position relative to the dimer, and the location of the entrance to the protein cavity. We also summarized the information about the number of ligand molecules bound by TFRs. Some regulators bind a single ligand molecule per dimer, while others can bind 2 or even multiple ligands simultaneously with different stoichiometry and affinity. Additionally, the structures of TFR complexes with different effectors were analyzed. The conformational changes upon binding different ligands by a particular TFR were minimal, even for the proteins with ligand-binding pockets that are particularly capacious and able to accommodate the effector at various locations. Such cavities exhibited substantially greater variability in the residues involved in the interaction with bound ligands. Furthermore, the TFRs representing a particular subfamily and interacting with the same ligand showed strikingly similar architecture and conformation of their CTDs, while TFRs from different subfamilies recognizing the same ligand differed substantially concerning the overall fold of their CTDs and the location of the ligand-binding cavity. The proposed classification accurately reflects sequence similarity and structural organization of the TFR CTDs [8]. Recently, we reported the first crystal structure of *Escherichia coli* CecR, a TFR related to cephalosporins and chloramphenicol resistance [10]. According to the proposed classification [8], this TFR belongs to subfamily D. Structural analysis revealed that indeed, it contains additional structural elements in its CTD, like helix α8a and the succeeding extended loop. The long tunnel-like ligand-binding cavity of CecR also seems to be characteristic for TFR subfamily D [10].

Our previous analyses on TFRs [8] reflect the enormous diversity of receptor proteins from that family. However, still each of the subfamilies includes thousands of members, and further studies of known but poorly described TFRs are necessary to make the characterization of newly identified TFRs easier. This motivated us to proceed to the next stage of research on TFRs—searching for sequential motifs underlying regulator–effector interactions. In this study, such sequence patterns were denoted as functional motifs, whereas other conserved motifs, not involved in interactions with effectors, were assigned as structural. Finding and examining sequence motifs conserved, especially the functional ones, among some members of the particular TFR subfamilies will enable function and effector prediction for many uncharacterized TFRs.

## Materials and Methods

### Identification of motifs characteristic for the TFR CTDs of different subfamilies

Identification of sequence motifs in the TFR CTDs, characteristic for the subgroups in each analyzed protein subfamily, was performed using a 2-step approach involving a conventional motif discovery using MEME Suite (version 5.4.0) programs and a heuristic machine-learning identification and selection of per-subfamily characteristic motif identification. As the NTD is highly conserved among TFRs, the sequences of all proteins were truncated by the first 50 residues. Keeping the division of the analyzed proteins into subfamilies from our previous work [8] and using the same set of sequences (InterPro superfamily: IPR001647), sequence motifs on the truncated sequences were recalculated using XSTREME [11] with the protein alphabet, minimum and maximum width of 6 and 15, respectively, *E*-value threshold of 0.05, clustering threshold of 0.05, and the background model of 0th Markov order. To ensure uniqueness of motifs, Tomtom [12] was additionally used, with a clustering threshold of *q* value at 0.05. The Tomtom results were later manually assessed and the motifs were additionally filtered.

To count the occurrence of each motif in sequences and score its fit, FIMO [13] from MEME Suite [14] was utilized. The background model used for FIMO was a 0th-order Markov model, and *q* value of 10^−4^ was used for cutoff. To address the imbalance between subfamily sizes, 300 sequences from each subfamily (the exception is subfamily I, which comprises only 104 sequences) were randomly selected. Output tabular data were read and transformed into a wide format using R (version 4.4.2) [15] and tidyverse library [16]. To ensure reproducibility, a seed was set on a model training step.

The taxonomic overrepresentation was controlled via pairwise similarity distribution evaluation using 30 sets of 300 random sequences selected from unclustered, and clustered at 90, 75, and 50% similarity subfamily sequences sets. The clustering was done using CD-HIT. Distribution similarity was evaluated visually using plots (Fig. S1) and statistics (Data S1), namely, Earth’s Mover Distance, Kolmogorov–Smirnov test and Cramer–von Mises test, and Mann–Whitney *U* effect size. Similarity statistics were calculated using scipy.stats (version 1.16.3) package in Python (version 3.12.12).

Further steps were carried out utilizing the R caret package (version 7.0-1) [17]. Selected sequences were split into training and testing sets in 3:1 proportion. Modeling was controlled using a repeated cross-validation method with 3 repeats over 3 folds. For the modeling purpose, each motif was treated as a separate feature, with FIMO score used as a numerical value for modeling. The score values were centered and scaled per predictor. We have tested 6 classification models—Decision Trees (rpart) [18], Random Forest (rf), Lasso and Elastic-Net Regularized Generalized Linear Model (glmnet) [19], k-Nearest Neighbors (knn), Partial Least Squares (pls), and Multi-Layer Perceptron (mlp). Only models that achieved 60% or better global accuracy on their best resample were considered further (rf, glmnet, pls, and mlp). The 60% cutoff was introduced to allow a heuristic selection of features that are common between models. The cutoff was introduced to avoid misidentification of the motifs as false negatives. The per-class (subfamily) importance of each predictor (motif) was extracted. Absolute values of importance were scaled to ensure comparability between models. Motifs that were characteristic for subfamily and common between models were further analyzed.

### Identification of the motifs in well-characterized TFRs

The motifs in TFRs of known function were identified using FIMO [13] from MEME Suite (version 5.4.0) [14]. The input sequences of 47 TFRs from our previous work [8] were used in this search. To minimize the possibility of false hit discovery, we have applied a stringent cutoff threshold of 10^−4^. Any hit on the list with a *q* value higher than the threshold was discarded. This produced a set of about 200 motifs, which were carefully checked. The location of each motif in the amino acid sequence of the well-characterized proteins was analyzed, and the motifs were divided into structural and functional ones. The motifs of the latter group contained residues involved in interactions with ligands. Ten well-known TFRs and their identified motifs (37 motifs in total) were selected for further analysis.

### Conservation of the residues within the selected motifs and analysis of the TFR homologs

The 37 conserved functional motifs found in 10 TFR representatives were used to search the sequences of the all-TFR dataset (InterPro superfamily: IPR001647), using FIMO [13] from MEME Suite [14]. As a result, 37 datasets with TFR sequences containing the searched motif were obtained (one dataset for each motif). For further analysis, one dataset and one corresponding motif per one analyzed TFR representative were used. The most specific motif of each TFR was chosen based on the following criteria: (a) the number of sequences in which the motif was identified should be ideally below 500, (b) it should be the motif identified in sequences belonging to only one TFR subfamily, and (c) the motif should contain residues important for interactions with ligands. The last condition was checked by structural analysis of the TFR–ligand complexes.

For the sequences containing the chosen motif, multiple sequence alignment (MSA) was calculated using MAFFT with default parameter settings [20]. This MSA and a structure of the analyzed TFR were used as the input for ConSurf analysis [21–23]. Furthermore, sequence logos [24], representing the occurrence of various residues in the motif, were prepared using a custom R script based on the ggseqlogo package [25]. Based on these sequence logos and MSA analysis in Jalview (version 1.8.3) [26], a Prosite [7] sequence pattern was proposed for each motif.

The information about the sequence origins for the sequences used in the MSA was downloaded from the UniProt database [27] and analyzed in regard to the bacterial families and species.

## Results and Discussion

### Identification of unique conserved motifs in the TFR CTDs

**Fig. 2.**
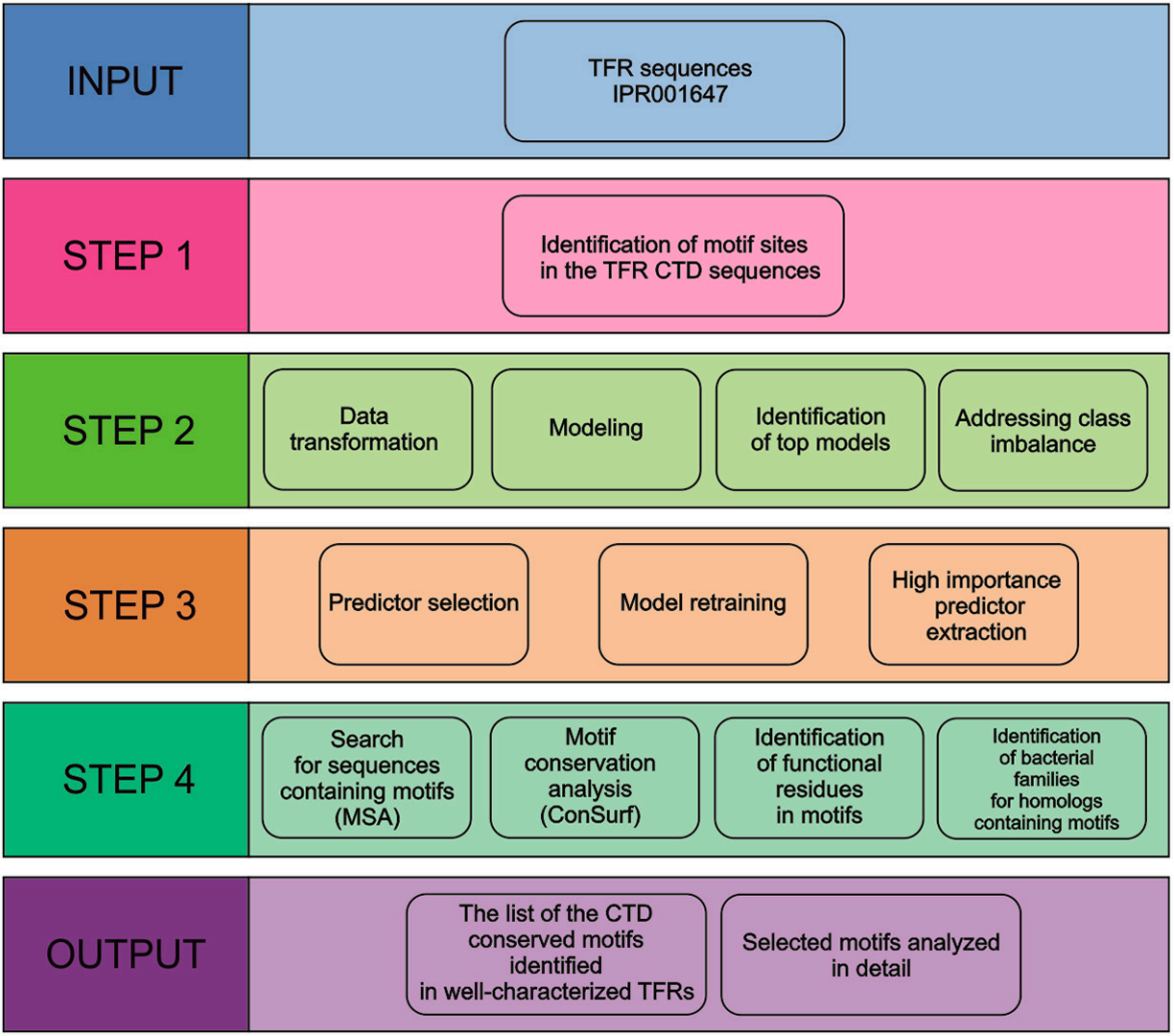
Workflow diagram presenting the analyses performed in our study. MSA, multiple sequence alignment.

To identify unique sequence motifs of TFR CTDs, the set of previously described [8] TFR sequences (InterPro ID: IPR001647) was used as an input. However, it is noteworthy that the sequences were truncated by the first 50 residues to remove the NTD, which is highly conserved among all TFRs. The heuristic machine-learning protocol was applied to identify the motifs characteristic for the subgroups in TFR subfamilies. The protocol included data transformation, modeling, identification of the top models, and addressing class imbalance followed by a validation process that comprised predictor selection, model retraining, and high importance predictor selection (Fig. 2). During the data preparation step, FIMO results were cleaned, and missing values were imputed as zeros, to ensure correct dataset shape. The dataset was then downsampled, split into training and testing sets, and used for initial modeling.

We have decided to use 4 approaches to classify the dataset: trees, linear models, distance-based model, and a neural network. We have tested 6 models: rpart [18], rf, glmnet [19], knn, pls, and mlp. Model selection was done based on overall accuracy. Only 4 models reached our criteria of at least 60% global accuracy: rf, glmnet, pls, and mlp. The subpar behavior of rpart and knn at this moment allows for insight into the dataset. The rpart tries to classify the dataset one feature at the time. This process resembles the construction of the simplified phylogenetic tree; instead of using the whole sequence, only parts are used for clustering. At the same time, the rf model, an ensemble of the trees, met our criteria. This could mean that the signal is too intricate for a single tree but manageable by an ensemble. Meanwhile, 2 linear models (glmnet and pls) succeeded while knn behaved subpar. That would indicate the linearity of classification; proteins containing specific motifs belong to the same family. However, the motifs can be degenerated; not all positions are conserved, which ultimately yields lower scores. That would position the entries from a single family on the line, but not necessarily, close to each other, in a cluster and in a multidimensional space. Additionally, the knn lacks feature selection. It is possible that we have used too many predictors and introduced noise, which interfered with distance-based classification. Since our goal was to identify the most characteristic sequence motifs among the subfamilies, we have selected the predictors that were common between the models used for classification. The selection of predictors included per-class importance extraction and intermodel scaling. The motifs found as most important for correct classification throughout selected models were further analyzed. It is worth noting that modeling was used as a tool for selection of the most characteristic motifs for further exploration, rather than for a definite classification.

After obtaining the set of conserved motifs, this set was cross-referenced against the 47 sequences of the well-characterized TFRs [8]. These proteins had been extensively characterized in our previous work [8], providing a substantial body of curated information. Most importantly, they also represent cases for which the structures of protein–ligand complexes are available. Since our aim was to identify sequence motifs encompassing residues directly involved in ligand interactions, access to such structural data was essential for reliable motif interpretation and validation. Several motifs were found in many of these TFRs, but not in all of them. The lack of conserved motifs in this small subset of TFRs might indicate that their sequence is highly unique, and their role is specific to only a few species that were characterized either by biochemical or only genomic studies. An example could be QacR, which was extensively studied over the years [28–30]; however, it was identified only in *Staphylococcus aureus* and differs substantially from the canonical TetR family members, e.g., it has a very large asymmetric ligand-binding pocket able to accommodate ligands at different sites and usually with stoichiometry: one ligand per dimer, which is rare for such a large cavity among TFRs [8].

The motifs identified in well-characterized TFRs were carefully analyzed, and it was checked whether the residues interacting with the ligands are contained in the motif. Based on this check, the motifs were divided into functional (involving residues recognizing ligands) and structural ones. The detailed list of the TFRs with corresponding motifs is presented in Data S2. All these motifs were also assembled as a .meme file (Data S3) that can be directly used as input in MAST [31] or FIMO [13] from MEME Suite [14] along with the sequence of the unknown TFR to verify whether it contains motifs present in known TFRs. This can be useful for the assignment of the potential function for newly identified and uncharacterized TFRs. It is of note that the *E* value should always be checked for each motif, and the lower the *E* value, the better the fit.

### TFR representatives selected for the detailed bioinformatic analysis

From all well-characterized TFRs in which conserved motifs were found, we selected 10 representatives (Table 1). The selected proteins belong to various TFR subfamilies and perform diverse biological functions. *Escherichia coli* TetR (Eco_TetR) is the best-characterized TFR protein that gave the name to the whole family and a canonical representative of subfamily K. It plays a role in antibiotic resistance and functions as a tetracycline repressor [33–35]. *Mycobacterium tuberculosis* (Mtu_EthR) is another TFR related to antibiotic resistance. It belongs to subfamily G and represses the *ethA* gene encoding a monooxygenase that activates the prodrug ethionamide used in tuberculosis treatment. Hence, the *ethR*-deficient strains are more sensitive to ethionamide treatment. This TFR became a target of many studies [37,38]. Three of the selected TFRs are related to multidrug resistance, *Salmonella enterica* RamR (Sen_RamR) [43–45], *Enterobacter lignolyticus* EilR (Eli_EilR) [36], and *Streptomyces lividans* EbrA (Sli_EbrA). Sen_RamR represents subfamily N, whereas Eli_EilR and Sli_EbrA belong to subfamily C. These regulators bind a wide range of ligands, including berberine, ethidium, crystal violet, malachite green, and proflavine. Additionally for Sen_RamR, bile acids, such as cholic and chenodeoxycholic acids, were also detected in its binding site [45]. *Pseudomonas putida* TtgR (Ppu_TtgR) is an example of TFR originating from plant-symbiotic bacteria. It belongs to subfamily E and binds plant-derived compounds, such as flavonoids (naringenin and quercetin) and polyphenols (phloretin and resveratrol), but also antibiotics (tetracycline and chloramphenicol) [41,42]. Two further TFRs, *Bacillus subtilis* FadR (Bsu_FadR) [32] and *Thermus thermophiles* FadR (Tth_FadR) [46], are related to fatty acid metabolism, but they are not orthologs, as they originate from evolutionarily distant bacterial species and belong to different TFR subfamilies (Bsu_FadR—E and Tth_FadR—L). *Pseudomonas aeruginosa* DesT (Pae_DesT) is another TFR related to fatty acid metabolism, but it controls the ratio of unsaturated to saturated fatty acids available for membrane-lipid synthesis [40]. It was classified as a subfamily N member. Finally, *Mycobacterium tuberculosis* KstR2 (Mtu_KstR2), belonging to subfamily E, functions as a regulator of the last steps of cholesterol degradation [39].

**Table 1.** TetR family regulators of which conserved sequence motifs are analyzed in the article

Protein	TetR subfamily	UniProt ID	Organism	Full name	Biological role	Ligands from the PDB structures	References
Bsu_FadR	EI	P94548	*Bacillus subtilis*	Fatty acyl-CoA dependent transcription factor	Fatty acid metabolism	Dodecyl-CoA and stearoyl-coenzyme A	[32]
Eco_TetR	K	P0ACT4	*Escherichia coli*	Tetracycline repressor	Antibiotic resistance	Tetracycline and its derivatives	[33–35]
Eli_EliR	C	E3G817	*Enterobacter lignolyticus*	Multidrug-binding repressor	Multidrug resistance	Crystal violet and malachite green	[36]
Mtu_EthR	G	P9WMC1	*Mycobacterium tuberculosis*	Transcriptional regulator EthR	Antibiotic resistance	Sutezolid and linezolid	[37,38]
Mtu_KstR2	EB	P9WMB9	*Mycobacterium tuberculosis*	Transcriptional repressor KstR2	Cholesterol degradation	Cholesterol-CoA derivatives	[39]
Pae_DesT	N	Q9HUS3	*Pseudomonas aeruginosa*	Transcriptional repressor DesT	Fatty acid metabolism	Palmitic acid and coenzyme A	[40]
Ppu_TtgR	EA	Q9AIU0	*Pseudomonas putida*	Transcriptional regulator TtgR	Regulation of efflux pump	Phloretin, naringenin, quercetin, tetracycline, resveratrol, and chloramphenicol	[41,42]
Sen_RamR	N	Q8ZR43	*Salmonella enterica*	Multidrug-resistance regulator RamR	Multidrug resistance	Berberine, ethidium, crystal violet, rhodamine 6G, dequalinium, and bile acids	[43–45]
Sli_EbrA	C	Q79SH7	*Streptomyces lividans*	Transcriptional repressor EbrA	Multidrug resistance	Ethidium, malachite green, and proflavin	n/a
Tth_FadR	L	Q5SM42	*Thermus thermophilus*	Fatty acyl-CoA dependent transcription factor	Fatty acid metabolism	Lauric acid and dodecyl-CoA	[46]

CoA, coenzyme A; n/a, not available; PDB, Protein Data Bank

All 37 motifs identified in the selected TFRs are listed in Table 2 and collected in .meme file (Data S4). We performed an additional search with these motifs against the whole TFR sequence set, and the results showed that many of these motifs were found exclusively in one subfamily to which the protein was assigned. However, in the case of 13 motifs (Figs. S2 and S3), they were additionally identified in subfamilies other than the assigned one. For TFRs belonging to the largest subfamilies E and K, the further classification into sub-subfamilies, according to Filipek et al. [8], was included during analysis (EA-EI and KA-KC). For most of them, there were only a few sequences out of the main subfamily, like FadR-1, TetR-3, or EilR-4 (Fig. S2). However, there are exceptions like motifs TetR-1 and EbrA-1 (Fig. S3), which are widely spread among various subfamilies. It is worth noting that while the presence of a single motif in a sequence may be an indicator of the protein belonging to the corresponding family, it does not determine its subfamily membership. The presence of multiple characteristic motifs is required for subfamily assignment.

**Table 2.** The analyzed motifs of the selected TetR family regulators

Protein	Family	Motif ID	Consensus motif	Matched sequence	Motif positions	*q* value	Number of identified sequences
Bsu_FadR	EI	FadR-1	EDILISLFZE	EDILISLFKE	51–60	5.29E−08	936
Bsu_FadR	EI	FadR-2	LAIVTQLELRQSNKE	LAIVTQLELRQSNLE	100–114	3.75E−13	1,269
Bsu_FadR	EI	FadR-3	INEVLKGYL	INEILKGYL	119–127	1.43E−07	278
Bsu_FadR	EI	FadR-4	MIFGAJDET	MIFGTIDET	155–163	2.37E−07	679
Eco_TetR	K	TetR-1	DREALLAALAAZGFE	NKRALLDALAVEILA	47–61	2.40E−05	11 305
Eco_TetR	K	TetR-2	NHTHSVPRAG	HHDYSLPAAG	63–72	4.07E−06	192
Eco_TetR	K	TetR-3	ALLAHRDGARVVAGT	ALLRYRDGAKVHLGT	89–103	2.51E−11	3 098
Eli_EilR	C	EliR-1	RLHAMLGSEDG	QLFSALGSEDG	82–92	3.98E−07	282
Eli_EilR	C	EliR-2	DGGFEPYIKLWREAQ	DGRLEPYIRLWRQAQ	91–105	5.80E−14	288
Eli_EilR	C	EliR-3	PHIKDAYLLTMZMWH	PEIKSAYLLTMNLWH	112–126	5.44E−13	372
Eli_EilR	C	EliR-4	AWRLIALVCGLDGIY	AWRLISLVCGLDGIY	152–166	1.06E−14	412
Mtu_EthR	G	EthR-1	WRNGIEAFFETFGSH	WRTGINVFFETFGSH	103–117	2.87E−15	442
Mtu_EthR	G	EthR-2	WSGFMQKWIDHTAAV	WSTFMQKWIAYTAAV	138–152	5.01E−15	392
Mtu_EthR	G	EthR-3	PARDLATALNWMNER	PAHELATALNLMNER	167–181	3.80E−13	888
Mtu_EthR	G	EthR-4	AIDTLAHIWLRSJYG	VLDTLVHIWVTSIYG	199–213	1.88E−11	1,270
Mtu_KstR2	EB	KstR2-1	STFLDELWAGYDAVL	RGFLDWLFARYRDIV	63–77	6.27E−07	1,121
Mtu_KstR2	EB	KstR2-2	AVAIYQREMHHL	QVVIYQDEAQRL	104–115	3.42E−06	2,301
Mtu_KstR2	EB	KstR2-3	EERNRZQRK	EDRNKQQRK	125–133	5.28E−07	165
Mtu_KstR2	EB	KstR2-4	DLDVRLTY	DLDVDLVY	151–158	6.56E−06	191
Mtu_KstR2	EB	KstR2-5	YRFIRDTVW	YRFIRDTTW	158–166	1.36E−08	1,505
Pae_DesT	N	DesT-1	ITSDLAADLARMPKL	ITDDLAADLALLNKM	132–146	9.69E−10	898
Pae_DesT	N	DesT-2	VRQLRMIAKGAYHWR	THQLRFIMIGGKHWH	191–205	3.82E−07	2,658
Pae_DesT	N	DesT-3	LKHWQGLG	GKHWHGLP	201–208	4.94E−05	296
Ppu_TtgR	EA	TtgR-1	EPDPLGCMRKLLIHL	EVDPLGCMRKLLLQV	82–96	8.68E−12	769
Ppu_TtgR	EA	TtgR-2	RRVFEILFHKCEFTD	RRINEILHHKCEFTD	107–121	7.04E−14	3 002
Ppu_TtgR	EA	TtgR-3	CDLRQQRQT	CEIRQQRQS	124–132	1.61E−06	466
Sen_RamR	N	RamR-1	KDELJNALYLHLKAD	KDELINTLYLHLKQD	51–65	5.96E−14	1,422
Sen_RamR	N	RamR-2	PKENARNIWNSYIDW	AKMMTRFIWNSYISW	81–95	5.74E−06	200
Sen_RamR	N	RamR-3	HRAJRQLAV	HRAIRQLAV	103–111	1.85E−08	151
Sen_RamR	N	RamR-4	MFPELRELCHRS	MFPELRDLCHRS	126–137	1.02E−12	303
Sen_RamR	N	RamR-5	SDEARAFGD	SDEYRAFGD	144–152	2.51E−08	169
Sen_RamR	N	RamR-6	QAERYRKSGFEAFWH	RAGEYIALGFEAMWR	172–186	8.28E−05	271
Sli_EbrA	C	EbrA-1	TKDELLAAALEHVAE	TLDDLMVAALRQANE	51–65	1.06E−08	9,607
Sli_EbrA	C	EbrA-2	GFAKVVAARGALEDP	GFARVVAAHPALSDP	66–80	9.63E−12	287
Sli_EbrA	C	EbrA-3	DRTGVELEY	DRTGVELEY	99–107	6.61E−08	301
Tth_FadR	L	tFadR-1	PYKEAVLLDYG	PYKEAVLLDYG	52–62	2.54E−10	85
Tth_FadR	L	tFadR-2	YELLNPDPVRAKAAF	YELLNPDPVRARAAF	110–123	5.01E−15	85

**Fig. 3.**
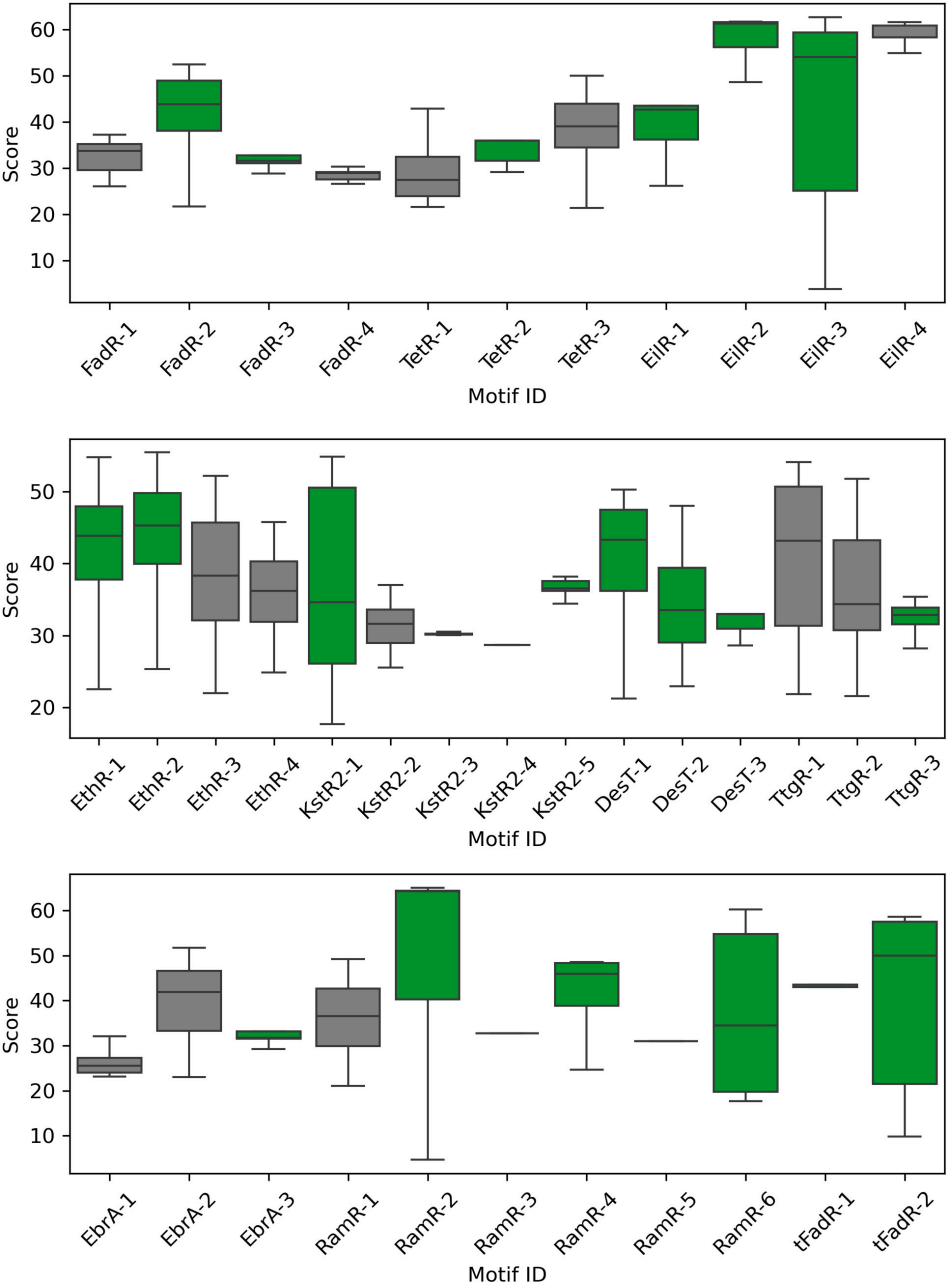
The calculated score distribution for sequence sets in which the analyzed motifs were found. Box-and-whisker plots are used to present the score distribution divided in quartiles; the whisker ends represent the minimum (Q0, 0th centile) and maximum (Q4, 100th centile) score values excluding outliers; the median is the black line in the box. The box colors, green and gray, present the motifs found exclusively in one TetR subfamily and the motifs found in several subfamilies, respectively.

The calculated motif score distribution for sequence sets in which the analyzed motifs were found is presented in Fig. 3. The FIMO score describes the log-likelihood ratio of the sequence containing the currently searched motif. The higher the score, the more probable that the identified motif hit was truly a positive match. It is worth noting that raw scores are a per-position sum of log likelihoods, and as such are length dependent, and longer motifs tend to have higher scores—10-residue-long motifs have medians around 30, and most of the 15-residue-long motifs present medians above that, even in the 50 to 60 range. Additionally, the score is affected by composition bias of the motif. Motifs with less-likely composition, in comparison to test sequence set residue frequency, will also have higher scores, which adds an additional layer to the analysis. However, the comparison focuses not on the absolute values of scores but rather on their distribution to show the variance and complements functional motif analysis. Low score dispersion indicates high conservation of the motif in its unchanged form. On the other hand, higher dispersion shows the flexibility of the sequence that can still be considered a hit, even after *q* value filtering. As the subfamily allocation was done by phylogenetic tree partitioning into clades, it would seem that the intrafamily motif scores should be less dispersed due to general sequence similarity. However, it is not the case, as both inter- and intrafamily motifs have high- and low-dispersion representatives.

Examples of low-dispersion intrafamily motifs are FadR-3, TetR-2, EilR-1, EilR-2, DesT-3, TtgR-3, EbrA-3, KstR2-3, KstR2-4, KstR2-5, and tFadR-1. On the other hand, there are high-dispersion intrafamily motifs like FadR-2, EilR-3, EthR-1, EthR-2, KstR2-2, DesT-1, DesT-2, RamR-2, RamR-6, and tFadR-2. Most of them are 15 residues long with a median score of about 40, indicating good, albeit not perfect, fit. The exceptions are DesT-2 (a 15-residue-long motif with a median score between 30 and 35), Ramr-6, and tFadR-2. In the case of DesT-2, the high dispersion and relatively low median indicate higher variability in some positions. The situation of RamR-6 and tFadR-2 is more interesting as they are 10 and 11 residues long with medians at 30 and 50, respectively. Additionally, the maximum observable scores for both motifs are around 60. This means that the composition of both motifs is highly unusual in comparison to the composition of the test set.

Among the low-dispersion interfamily motifs, there are FadR-1, FadR-4, TetR-1, EilR-4, KstR2-2, and EbrA-1. As stated, the share of sequences from other families in the so-called interfamily motifs is miniscule, with 2 exceptions—TetR-1 and EbrA-1. Despite being 15 residues long and having very low score dispersion, both motifs present a median between 25 and 30. The other end of the interfamily spectrum is the EilR-4 motif, a 15-residue-long fragment with low dispersion and median around 60, indicating very high conservation. Interestingly, the only other motif that has a median around 60 and relatively low dispersion is EilR-2, also from the multidrug-binding EilR protein. The mentioned motifs form the binding-site cavity [36].

### Motif conservation analysis and identification of functional residues in the motifs

The selected motifs were further analyzed (Fig. 2, step 4) to check the residue conservation within the motifs. For this purpose, the TFR sequence sets in which the motifs were identified were utilized. As we decided to use the ConSurf server [21–23], the MSAs were calculated for the sequence sets. The ConSurf calculations were possible only for MSA that did not include more than 500 sequences. Thus, for each TFR, a set fulfilling these requirements was chosen. For instance, in the case of Bsu_FadR, a set of sequences with the FadR-3 motif was used, as only the size of this set (with 278 sequences) was appropriate. The number of sequences found for each motif is listed in Table 2.

**Fig. 4.**
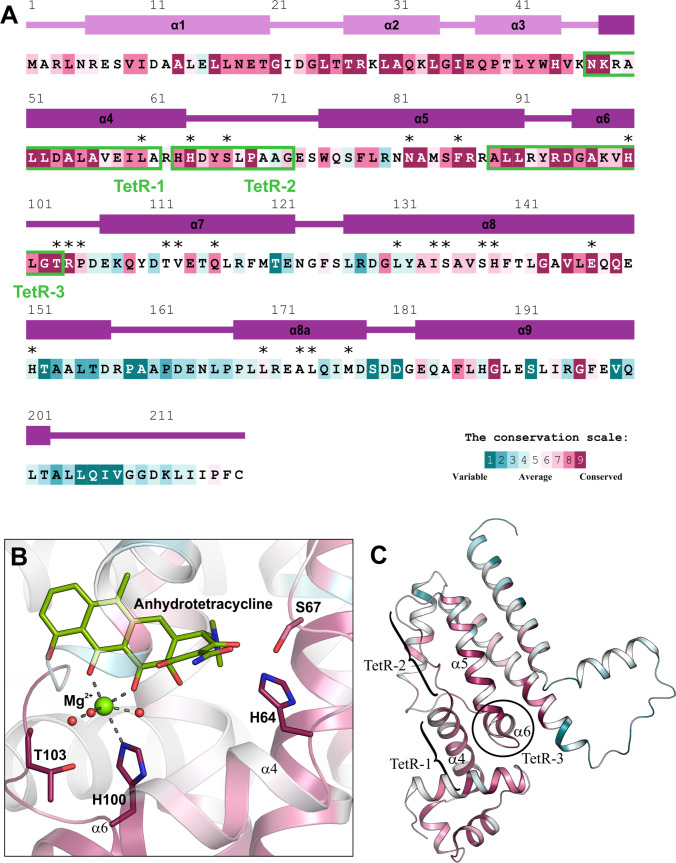
The ConSurf analysis of TetR performed using the crystal structure of Eco_TetR (PDB ID: 4D7M) and MSA calculated for the sequence set with identified motifs. (A) The amino acid sequence of Eco_TetR is colored according to the conservation score calculated by ConSurf. The secondary structure elements are numbered and shown above the sequence. The asterisks indicate the residues interacting with ligands in all known Eco_TetR structures. (B) Ligand-binding site of Eco_TetR. For clarity, only the residues present in the identified motifs and interacting with the presented ligand are shown as sticks. The magnesium ion and water molecules coordinating it are displayed as green and red spheres, respectively. The helices of chain A are colored according to the ConSurf scale, whereas chain B is presented in light gray. (C) Cartoon representation of chain A of the Eco_TetR dimer with the highlighted motifs.

Using the results from the ConSurf analysis, we have mapped the identified motifs on the sequences and structures of the TFR representatives, together with the residues participating in the interactions with ligands (Fig. 4A and B; Figs. S4 to S12A and B). The great majority of residues interacting with ligands and present in the motifs are conserved. For instance, in Eco_TetR all these residues are highly conserved (Fig. 4A and B). H64 and S67 (of the TetR-2 motif) localized in the long loop between α4 and α5 form hydrogen bonds with tetracycline (and its derivatives), whereas H100 (of the TetR-3 motif) coordinates magnesium ion, usually bound with tetracycline, and T103 interacts with water molecules of the magnesium coordination sphere. Both residues, H100 and T103, are highly important for the Eco_TetR response to tetracycline presence, as the movement of their side chains toward magnesium ion (or tetracycline in the absence of divalent ion) triggers the conformational changes of this regulator [35]. Hence, our search highlighted motifs that include residues important not only for protein–ligand interaction but also residues actively participating in conformational changes occurring in the regulator structure. Similarly, in Bsu_FadR (Fig. S3A and B), almost all interacting residues in the motifs are highly conserved and R109 (in the FadR-2 motif) is a key residue involved in conformational changes of this regulator [32]. Interestingly, in Tth_FadR (Fig. S5A and B), some of the interacting residues of the motifs exhibit high conservation, whereas others have only average conservation scores. It is another evidence that the 2 FadRs are evolutionary distant. High conservation of the analyzed residues was detected in Mtu_KstR2 (Fig. S6A and B). It is of note that in both Tth_FadR and Mtu_KstR, large regions of the CTDs are highly conserved. This might be partially linked to the fact that a relatively small number of sequences was used for MSA in these cases (Tth_FadR—85 sequences; Mtu_KstR2—165 sequences) compared to other analyzed sequence sets. In Mtu_EthR (Fig. S7A and B), the residues interacting with ligands (present in the motifs) are also conserved (but often not with the highest scores) with few exceptions that are in the average conservation range. Two of the analyzed residues (W138 and W145 of the motif EthR-2) are involved in large-scale movements related to conformational changes in this regulator [38]. The residues interacting with ligands (in motifs) in Pae_DesT (Fig. S8A and B) also represent high conservation scores (with 2 exceptions). Interestingly, the conserved motifs of this regulator were mapped close to the C terminus of the protein. Two of the 3 identified motifs are localized at helix α9 and the highlighted residues interact with coenzyme A, in contrast to other above analyzed TFRs, where the residues in the motifs showed rather hydrophobic interactions with fatty acid chain, like FadRs or Mtu_EthR. The conservation of the analyzed residues in TFRs interacting with multiple and variable ligands like Eli_EilR (Fig. S9A and B), Sli_EbrA (Fig. S10A and B), Sen_RamR (Fig. S11A and B), and Ppu_TtgR (Fig. S12A and B) is usually a bit lower compared to metabolic regulators. Especially in Eli_EilR and Sli_EbrA, the conservation score of some residues is more often average or even lower compared to other analyzed TFRs.

**Fig. 5.**
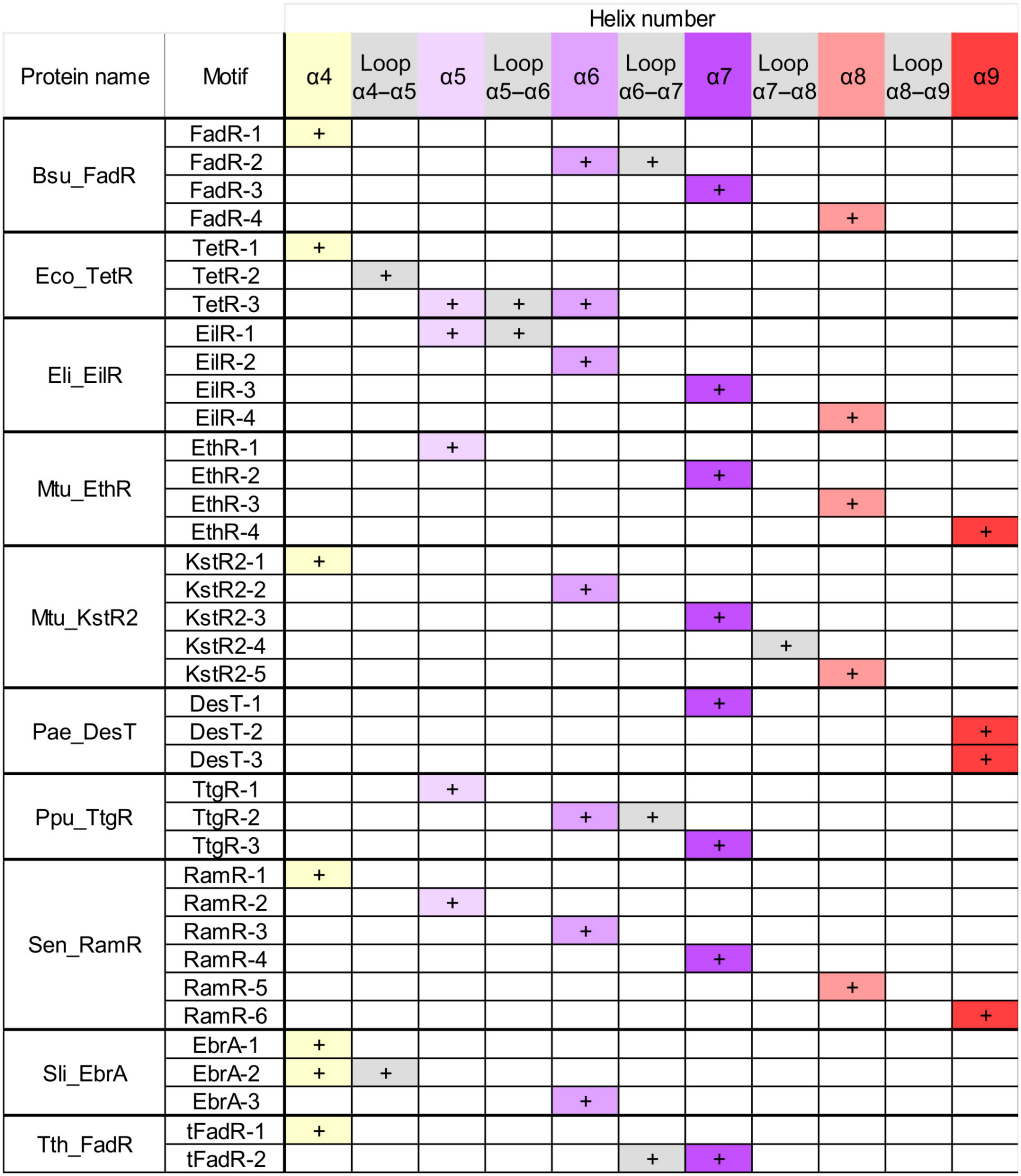
Matrix presenting location of the analyzed motifs in the CTD structural elements. The helices of CTD are colored according to their functions (as in Fig. 1): helix α4 (conformational changes)—yellow; helices α5 to α7 (the central triangle of CTD)—purple; helices α8 to α9 (dimerization)—red. The loops are shown in gray.

We further checked the location of the motifs in the crystal structures of TFRs (Fig. 4C and Figs. S4 to S12C). The results of this analysis were collected in the form of a matrix (Fig. 5). Among the analyzed proteins, the conserved motifs are located predominantly on the helices. However, some motif parts are extended to the loop regions. Interestingly, the TetR-2 motif is entirely present in the long loop between helices α4 and α5 (Fig. 4C). The great majority of the identified motifs are present on helices α5, α6, and α7 that form a central triangle of the TFR CTDs and often participate in the formation of the ligand-binding cavity. Some TFRs, like Mtu_EthR or Sen_RamR, have conserved motifs located on helices α8 and α9, which are associated with dimerization (Fig. 5). In these proteins, the residues of these helices are involved in ligand-binding cavity formation. A very special case is Pae_DesT that has a large cavity with the entrance located on the top of the dimer and formed mainly by helix α9 (Fig. S8C). For 6 out of 10 analyzed TFRs, at least one conserved motif was identified in helix α4. These motifs indeed contain residues participating in interactions with ligands but represent low specificity and were usually found in the sequence sets containing above 1,000 sequences. An exception is tFadR-1 identified in only 85 sequences. This proves that it is a very unique TFR feature. Additionally, the motifs TetR-1 and EbrA-1 (found in about 10,000 sequences) were also widely distributed among various subfamilies (Fig. S3). Thus, the motifs present in helix α4, in most cases, seem not to be highly specific for a particular TFR and should not be used for the identification of the TFR role. It is in good agreement with previous studies, as it was shown that in addition to the NTD motifs, it is possible to find conserved sequence patterns characteristic for the whole TFR family also in helix α4 [47]. Notably, α-helices present at the domain interfaces often play key roles in stabilizing protein structure and mediating conformational changes, as also proved for other proteins [48–51].

### Motifs of the selected TFRs translated into the Prosite signature

The prepared .meme files with conserved motifs (Data S3 and S4) enable detailed analysis of the uncharacterized TFRs. However, we thought that it might also be useful to propose a Prosite pattern for the analyzed TFRs. Such a pattern would also allow for the identification of putative TFRs that recognize the same type of ligands and most likely are involved in the same regulatory role. As the PROSITE database [7] is an important bioinformatic resource, such Prosite patterns might improve functional annotation of the putative receptor in future.

**Fig. 6.**
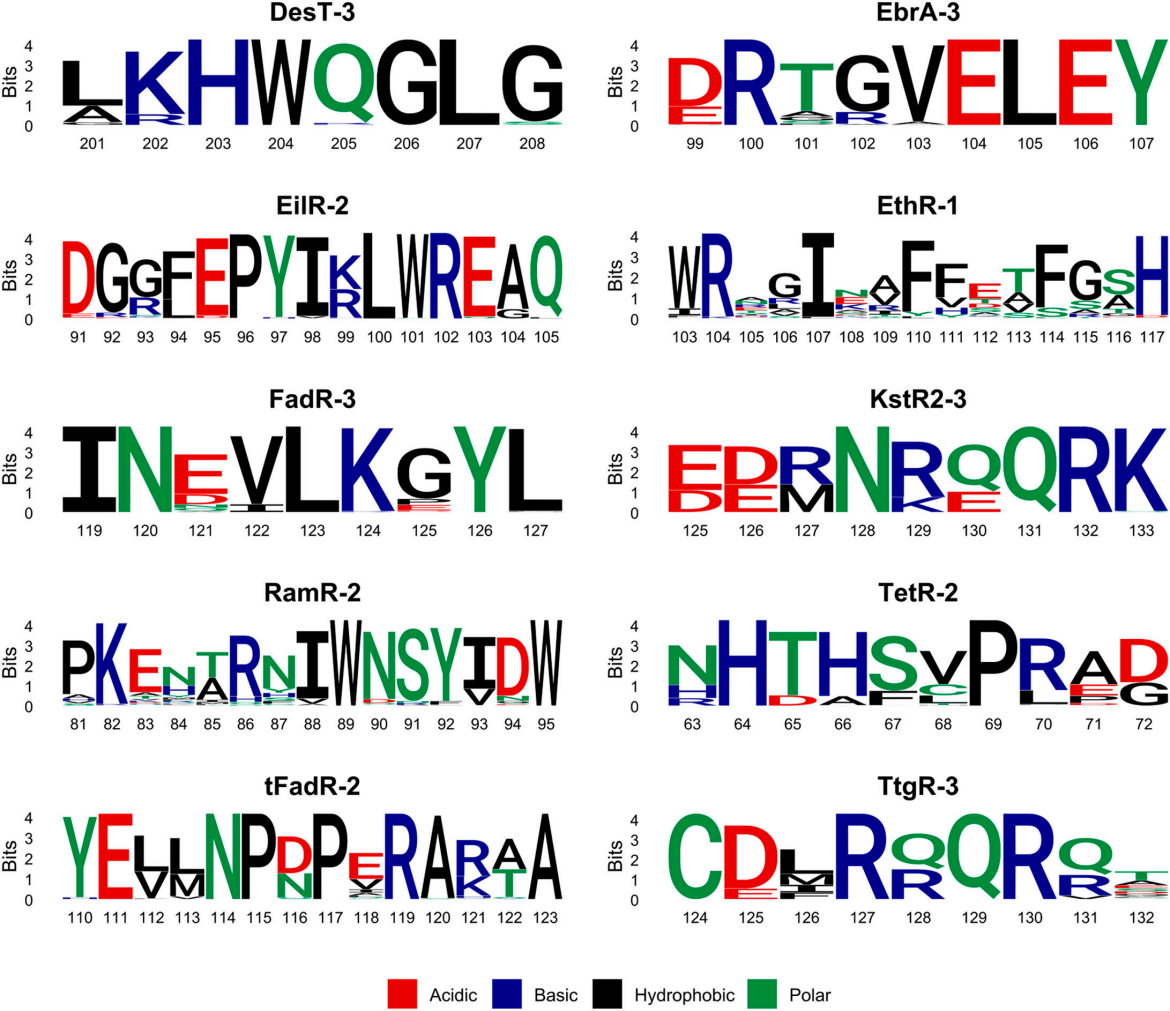
The sequence logos representing the conservation score and compositional variability at each position of the selected motifs, created based on the aligned set of sequences analyzed with ConSurf. The *y*-axis shows the bit score, where a maximum score of ~4.3 for proteins represents a 100% conservation. The lower the cumulative height of a letter stack reflected by a lower bit score, the bigger the tolerance for substitutions at that position. The relative sizes of amino acids within each stack represent the percentage-wise share of that residue in that position in all aligned sequences, with a dominant amino acid placed on the top of the stack. The *x*-axis displays the position of a corresponding amino acid in the TFR from which the motif originates. The amino acid residues were colored according to their chemical properties.

For this purpose, we decided to choose one representative, specific motif per each TFR. This selection was done based on several criteria: (a) the number of sequences in which the motif was identified should not be too high, ideally below 500 (as in MSA for ConSurf); (b) it should be the motif identified in sequences belonging to only one TFR subfamily; and (c) the motif should contain residues important for interactions with ligands, ideally more than 1 or 2. For each of the selected motifs, a sequence logo was generated (Fig. 6). The analysis of sequence logos reveals a set of characteristics shared between selected motifs, as well as more pronounced differences between them. Nine out of 10 of these motifs have at least one conserved hydrophobic residue in their sequences, such as the FadR-3 motif with I119, L123, and L127, or variable positions at which only hydrophobic substitutions are allowed, such as positions 112 and 113 in the tFadR-2 motif or position 126 in TtgR-3. These hydrophobic amino acids are either buried, contributing to the folding of the protein, or are located within the inner surface of the binding cavity to participate in protein–ligand interactions. The clear exception from this rule is the KstR2-3 motif, in which there is only 1 out of 9 positions, where a nonpolar amino acid, methionine, is detected in some of the aligned sequences, yet it can be substituted to a positively charged arginine with equal frequency. All other amino acids in the KstR2-3 motif are polar or charged. Conserved non-hydrophobic amino acids or positions dominated by polar, acidic, or basic substitutions are also common in analyzed motifs. Based on the location of the motif in structures, they are usually exposed to the solvent, while in some specific cases they either interact with other proximal non-hydrophobic side chains, like the C-terminal H117 from the EthR-1 motif, or with polar functional groups of ligands, such as the H64 from the TetR-2 motif (Fig. 4B). Substitutions of amino acids of vastly opposite chemical characters at certain positions in the motif sequence also appear in all 10 analyzed motifs. Motifs EthR-1 and RamR-2, which are relatively long compared to some other analyzed motifs, are constructed mostly of these generally variable positions with multiple substitutions of different chemical characters. Other motifs typically have no more than a couple of allowed amino acid substitutions within each position. Finally, by looking at these 10 sequence logos, the last particularly interesting and important observation can be made—each motif is unique, and both the conservation and chemical character of its amino acids need to be assessed separately and in the context of structures of proteins in which it is present. Moreover, this uniqueness of analyzed motifs is an additional sign of their functional and subfamily-level speciation. The only 2 motifs that share any surface-level similarities between their sequence logos are KstR2-3 and TtgR-3, for which at positions 129 to 132 and 127 to 130, respectively, a 4-residue-long submotif R-Q-Q-R can be detected, with the second glutamine and arginine being completely conserved in both motifs. This resemblance is not surprising, taking into consideration that these 2 motifs are derived from TFRs from the same subfamily E (Table 1). The 10 chosen conserved sequence motifs from the TFR and their sequence logos serve as a solid base to determine Prosite patterns.

Using sequence logos, supported with additional analysis of MSA in Jalview [26], where the detailed amino acid residue percentage share at each position can be checked, the Prosite pattern was proposed for each of the selected motifs (Table 3). To validate the proposed Prosite patterns, we performed a search of the sequences containing these patterns with the ScanProsite tool against the SwissProt database or Protein Data Bank during the search, the approximate number of expected random matches (ERMs) in ~100,000 sequences (50,000,000 residues) was calculated. The pattern for FadR-3 turned out to be highly specific and the ERM could not be obtained, as it was close to zero [52]. Reliable ERM and search results were also obtained for EilR-2, KstR2-3, RamR-2, and tFadR-2. Although ERM for TtgR-3 was 8.28e−02, a bit higher than for the other proper patterns, which did not give random hits, the search results included only Ppu_TtgR. Thus, we assume that it might be specific enough to serve as a valid signature. It is worth noting that, except for RamR-2 and tFadR-2, all the motifs had low score variance, and while in the case of RamR-2 and tFadR-2 the variance was high, the median was also high, indicating a population of highly specific motif hits.

**Table 3.** The Prosite patterns proposed on the basis of conserved sequence motifs

Motif ID	Proposed Prosite pattern	Approximate number of expected random matches in ~100,000 sequences (50,000,000 residues)	Prosite signature
DesT-3	[LAG]-[KR]-H-W-[QRH]-G-L-[GSP]	4.77e−02	NO
EbrA-3	[DE]-R-x(2)-V-E-L-E-Y	0.21	NO
EilR-2	[DE]-x(3)-[ED]-P-[YH]-[IV]-x-L-W-R-[EQ]-[AG]-[QL]	2.51e−05	YES
EthR-1	[WI]-R-x(2)-I-x(2)-F-x(3)-[FS]-x(2)-H	0.94	NO
FadR-3	IN-x-[VI]-LK-[GPE]-YL	n/a	YES
KstR2-3	[ED]-[DE]-[RM]-N-[RK]-[QE]-Q-R-K	2.97e−03	YES
RamR-2	K-x(3)-[RH]-x-[IV]-W-N-S-Y-[IV]-x-W	4.77e−05	YES
TetR-2	[NHR]-H-[TD]-[HAY]-[SF]-x-P-x-x-[DG]	1.1	NO
TetR-3	A-L-L-x-[YH]-R-D-[GD]-A-x(2)-H-x-G-T	3.82e−06	YES
tFadR-2	Y-E-x(2)-N-P-[DN]-P-x-R-A-x(2)-A	2.97e−04	YES
TtgR-3	C-[DE]-x-R-[RQ]-Q-R-[QR]	8.28e−02	YES

n/a, not available

In contrast, the other proposed patterns had a high ERM and a search with these motifs usually provided several unspecific hits, like enzymes and proteins not only from bacteria but also from plants. Thus, for these TFRs, we tried to use other motifs to generate Prosite patterns; however, it was successful only for TetR-3 (Fig. S13). In this case, the calculated ERM was low (3.82e−06) and the search gave only Eco_TetR homologs as hits. The reason for the inability to obtain proper patterns for other TFR motifs might be that these motifs were highly degenerated and at most of the positions several similar amino acid residues were possible. Yet, still such motifs in .meme matrices that include many possible residues with percentage probability allow for a reliable search with MEME Suite.

### Bacterial families of TFR homologs identified based on the CTD motifs

**Fig. 7.**
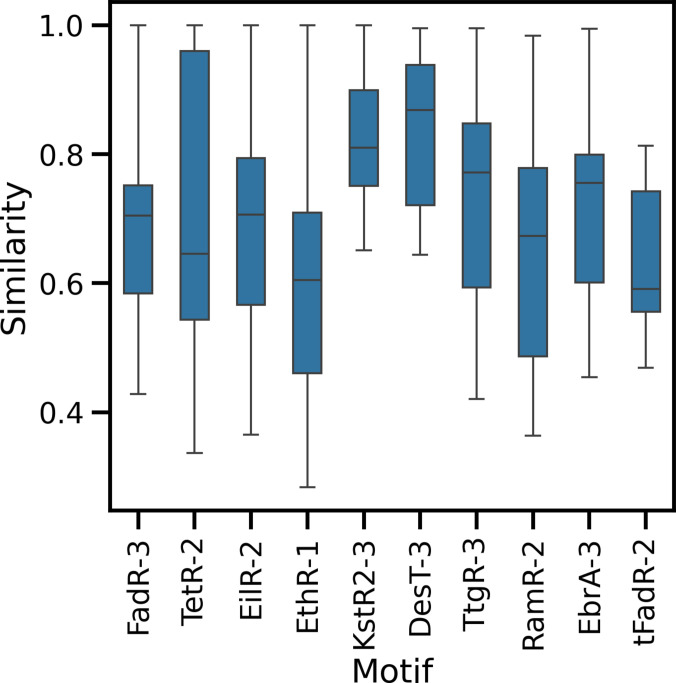
The similarity between the sequences containing the selected motifs. Box-and-whisker plots are used to present the similarity score distribution divided into quartiles; the box represents Q2 and Q3 (25th to 75th centiles), the median (50th centile) is the black line in the box, and the whisker ends represent the minimum (Q0, 0th centile) and maximum (Q4, 100th centile) score values excluding outliers.

In the last step, we examined the sequence sets in which the specific selected motifs were identified, further described as homologs of the analyzed TFRs. The similarity between each pair of the sequences containing the selected motif was calculated and plotted using the median score as reference for each set (Fig. 7). The highest similarity (above 60%) in the sequence set was observed for KstR-3 and DesT-3, whereas in almost all other cases, it varied from ~40% up to 100%. The lowest similarity values were observed in sequence sets of TetR-2, EilR-2, and EthR-1. We assume that the identification of the motifs in sequences with low similarity indicates that these TFRs might still recognize very similar ligands and regulate the same metabolic or stress-response pathways in bacteria.

**Fig. 8.**
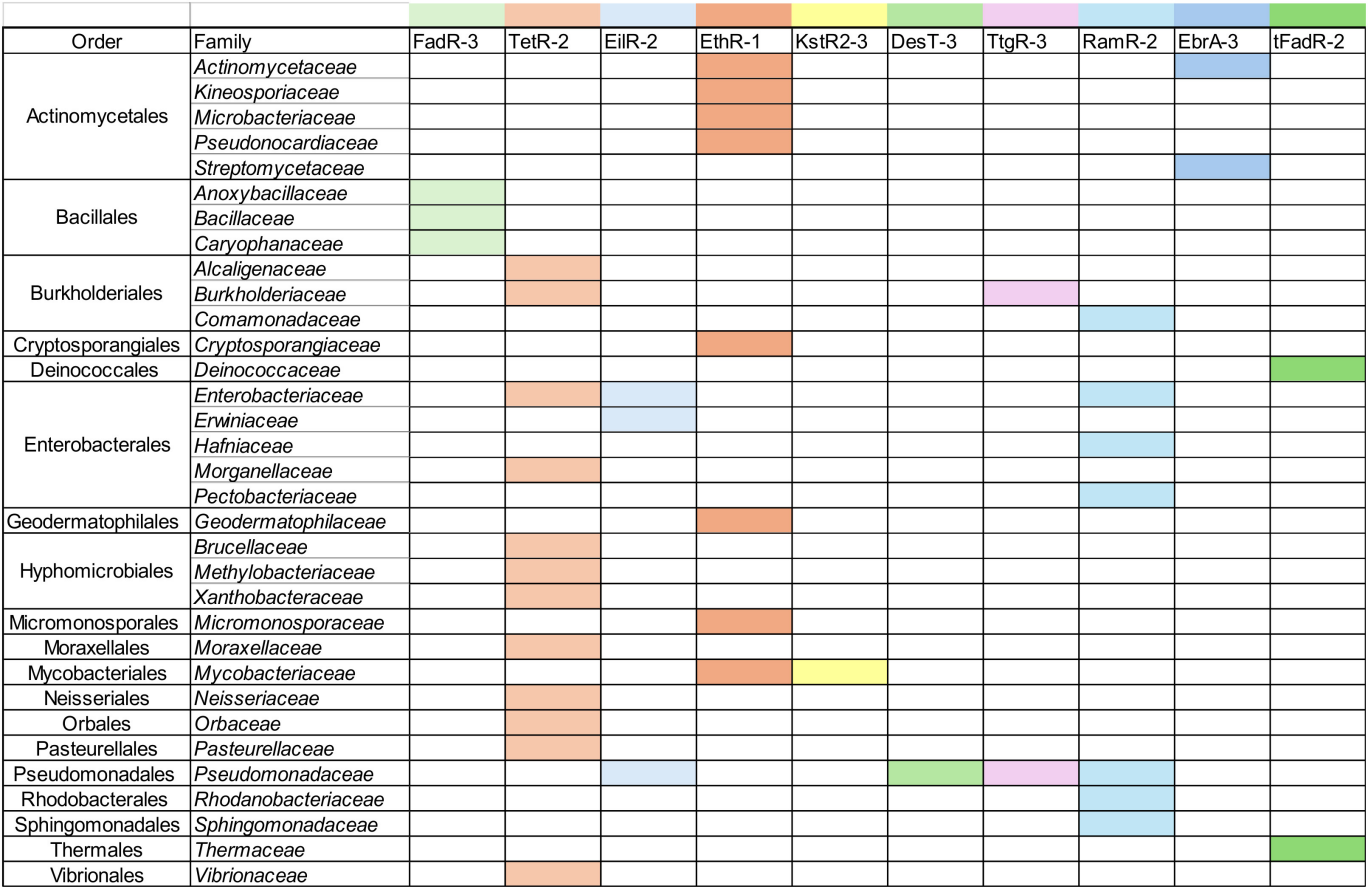
The distribution of the sequences with identified motifs among bacterial orders and families. The colors relate to the biological process in which the protein of the motif origin is involved: shades of red—antimicrobial resistance, shades of blue—multidrug resistance, pink—control of efflux pump in plant-related bacteria, shades of green—fatty acid metabolism, and yellow—cholesterol degradation.

Furthermore, we have revised the assignment of homologs to bacterial families. At this point, it is important to note that databases contain multiple records of extensively studied species and model organisms. Thus, we performed only a qualitative analysis of the identified bacterial families of the homologs because quantitative visualization could suggest an inaccurate picture. Therefore, the list of the motifs and the bacterial families of homologs, in which these characteristic motifs were found, was compiled in the form of a matrix (Fig. 8).

**Fig. 9.**
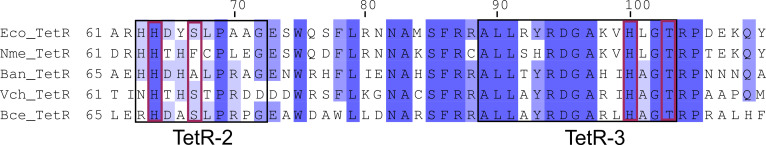
Conserved motifs in Eco_TetR homologs. The amino acid sequence of Eco_TetR was aligned with its homologs: *Neisseria meningitidis* TetR (Nme_TetR, UniProt ID: Q8L0M9), *Brucella anthropi* TetR (Ban_TetR, UniProt ID: A6X092), *Vibrio cholerae* TetR (Vch_TetR, UniProt ID: A0A7Z7VIJ5), and *Burkholderia cepacia* TetR (Bce_TetR, UniProt ID: A0A8I1AQ96). Sequences were visualized using Jalview. Intensity of the blue color gradient reflects sequence conservation. Numbering above the alignment is according to Eco_TetR. The TetR-2 and TetR-3 motifs are presented in black boxes. The residues present in the motifs and interacting with ligands are highlighted with magenta boxes.

The TetR-2 motif was detected in TFRs of bacterial families of the order Enterobacterales, such as *Enterobacteriaceae*, which includes *Escherichia coli*, and *Morganellaceae*. We also identified this motif in homologs from other bacterial orders. Specifically, it was found in members of the Hyphomicrobiales order, including the *Brucellaceae*, *Methylobacteriaceae*, and *Xanthobacteraceae* families, as well as the Burkholderiales order, represented by the *Alcaligenaceae* and *Burkholderiaceae* families. Additionally, we observed TetR-2 in homologs from bacteria belonging to the families *Moraxellaceae*, *Neisseriaceae*, *Orbaceae*, *Pasteurellaceae*, and *Vibrionaceae*, each representing a different order. All the indicated homologous sequences were found to originate from representatives of gram-negative bacteria, which proves the conservation and evolutionary importance of the TetR-2 motif in this type of bacteria, widely distributed outside the *Enterobacteriaceae* family. To verify whether this analysis can be useful for identifying of homologs exhibiting a similar ligand-recognition pattern, we chose 4 homologs of Eco_TetR, representing different bacterial families and identified in pathogenic bacteria, namely, *Neisseria meningitidis* TetR (Nme_TetR), *Brucella anthropi* TetR (Ban_TetR), *Vibrio cholerae* TetR (Vch_TetR), and *Burkholderia cepacia* TetR (Bce_TetR). Their sequence identity to Eco_TetR is 64%, 50%, 47%, and 43%, respectively, which indicates that these are not highly similar proteins. The conserved motifs could be easily detected based on the sequence alignment of Eco_TetR with the sequences of homologs. Our analysis focused on the 2 motifs, TetR-2 and TetR-3 (Fig. 9), which contained the residues important for interactions with tetracycline and magnesium ion. Both motifs were conserved across the analyzed sequences. As the conserved motifs directly contribute to the formation of the ligand-binding pocket, their preservation across homologous proteins strongly suggests a similar pocket architecture. In general, conservation at the sequence level typically translates into similar 3-dimensional structures, which in turn underpins similar mechanisms of action. Moreover, residues corresponding to H64, H100, and T103 of Eco_TetR were conserved in the analyzed sequences, whereas only S67 was not present in all homologs. This also implies comparable ligand-binding properties. Furthermore, the conservation of H100 and T103 triggering the conformational changes of Eco_TetR [35] suggests that it is very likely that the homologs exhibit similar regulatory mechanisms.

The conservative EthR-1 motif was found in homologous proteins of bacteria from the *Mycobacteriaceae* family, including *Mycobacterium tuberculosis*, and in other gram-positive bacteria. The identified Actinomycetales included the *Actinomycetaceae*, *Kineosporiaceae*, *Microbacteriaceae*, and *Pseudonocardiaceae* families, as well as representatives from other bacterial orders, including the *Cryptosporangiaceae*, *Geodermatophilaceae*, *Micromonosporaceae*, and *Mycobacteriaceae* families.

The next group of the analyzed TFRs represents the regulators involved in multidrug resistance. The list of homologs with the EilR-2 motif (from *Enterobacter lignolyticus* EilR) includes families from the orders Enterobacterales (*Enterobacteriaceae* and *Erwiniaceae*) and Pseudomonadales (*Pseudomonadaceae*). The EbrA-3 motif of EbrA from *Streptomyces lividans* was identified in the Actinomycetales order, specifically in the *Actinomycetaceae* and *Streptomycetaceae* families. Interestingly, although both regulators, EilR and EbrA, recognize the same type of ligands and belong to the same subfamily C, the EbrA-3 motif is characteristic of gram-positive bacteria, whereas the EilR-2 motif was found in gram-negative bacteria. This shows that even in the same TFR subfamily, further subgroups of the TFRs of similar function might be diverse due to bacterial origin. Another motif analyzed in the group of regulators linked to multidrug resistance is RamR-2 of RamR (subfamily N) from *Salmonella enterica*. While EilR-2 and EbrA-3 were identified only in homologs belonging to a few bacterial families, the RamR-2 is widely distributed among various bacterial families from different orders. The homolog sequences including the RamR-2 motif were found in the order Enterobacterales, specifically in the families *Enterobacteriaceae* (including *Salmonella enterica*), *Hafniaceae*, and *Pectobacteriaceae*, also in the orders Burkholderiales, Pseudomonadales, Rhodobacterales, and Sphingomonadales. Another very specific example of a regulator involved in recognition of various ligands is TtgR, which was characterized in plant-symbiotic bacteria *Pseudomonas putida* and binds flavonoids [41]. Thus, it is not surprising that among the homologs including the TtgR-3 motif, representatives of the *Pseudomonadaceae* family were almost exclusively found, with only one hit in the *Burkholderiaceae* family.

Another group of ligands recognized by TFR is represented by fatty acids. However, the conservative motifs FadR-3, tFadR-2, and DesT-3 that we have identified are not very similar, as all the analyzed TFRs in this group interact with different fatty acids or their derivatives and belong to different TFR subfamilies. FadR-3, which was characterized in FadR (subfamily E) from *Bacillus subtilis*, was identified in homologs belonging to the order Bacillales, represented by the families *Anoxybacillaceae*, *Bacillaceae*, and *Caryophanaceae*. The second motif, tFadR-2 of FadR (subfamily L) from *Thermus thermophilus*, was only found in the order Thermales, including the family *Thermaceae*, as well as in the order Deinococcales, which has representatives in the family *Deinococcaceae*. Homologs including the DesT-3 motif of DesT (subfamily N) from *Pseudomonas aeruginosa* were identified exclusively in the order Pseudomonadales, in the family *Pseudomonadaceae*.

The last analyzed motif was KstR2-3 of KstR2 from *Mycobacterium tuberculosis*, which is also involved in metabolism regulation and plays a key role in the process of cholesterol degradation [39]. Our analysis showed that KstR2-3 was exclusively found in homologous sequences belonging to the *Mycobacteriaceae* family, suggesting its high conservation within this taxon.

Based on this analysis, the most widely distributed motifs among various bacterial families are related to antibiotic or multidrug resistance. This observation is very promising in terms of development of novel antimicrobial factors, as one drug targeting TFR with homologs in many bacterial families will most likely have a broad spectrum of activity against various bacteria. In contrast, the motifs identified in TFRs involved in metabolism control are rather conserved only among small groups of closely related bacteria.

## Conclusion

In this study, we identified unique conserved sequence motifs of TFR CTDs using a machine-learning protocol. Furthermore, these motifs were found in well-characterized TFRs and collected in a form of a detailed list (Data S2) and as a .meme file (Data S3) that can be directly used for search of these motifs in newly identified TFRs, which enable the assignment of the TFR function. For initial characterization of a new uncharacterized TFR, we recommend a check of its amino acid sequence against motifs collected in Data S2 (a list of motifs linked to known TFRs) and Data S3 (a file to be directly used in MAST). The more motifs, assigned in Data S2 to a known TFR, will be found in the uncharacterized TFR sequence, the higher the chance of its proper function recognition. Moreover, we selected 10 well-characterized TFR representatives and analyzed in detail all motifs found in their CTDs. We checked the distribution of these motifs in TFR subfamilies and analyzed its overall conservation using the ConSurf server. This analysis showed that most of the residues involved in ligand binding present in the identified motifs exhibit high conservation scores, with some exceptions. Lower conservation scores for such residues were mainly observed in TFRs involved in multidrug resistance. Additionally, we mapped the localization of the conserved motifs in the structural elements of CTDs. The motifs were present not only in helices α5 to α7 of the central triangle but also in helices α4 or α8 and α9, depending on the location of the ligand-binding cavity in the CTD of a particular TFR. This finding highlights great variability of the TFR CTDs.

As the PROSTIE database is an important source of information about the conserved patterns and helps the functional annotation of the putative proteins, we also transformed the analyzed motifs into the Prosite patterns, which could be used for searching with Prosite tools. It is of note that the provided reliable Prosite signatures (Table 3) are based on a single highly conserved functional motif per TFR. Such a motif is sufficient for initial function prediction, as proved by a follow-up search of further conserved motifs in the uncharacterized TFR sequence, using Data S3 and MAST, which typically identifies further motifs implying the homology to the known TFR. Finally, we identified the homologs of 10 TFR representatives in the corresponding TFR subfamilies to find the proteins that likely play a similar role, compared to well-characterized TFRs, in bacteria. This analysis showed that motifs of TFRs related to antibiotic or multidrug resistance are broadly distributed among various bacterial families, which is an important finding in terms of the design of new antimicrobials. In conclusion, our results provide useful information, in the form of conserved sequence motifs, for future characterization of putative TFRs that is essential for developing new antimicrobial therapeutics.

## Data Availability

The most important data that support the findings of this study are included as supplementary materials. The custom scripts, full lists of the motifs, and sequences sorted by subfamilies are available at https://doi.org/10.34658/RDB.WJDR6Z.
