# Supplementary material for: Conserved Motifs in the Ligand-Binding Domain of TetR Family Regulators: Identification and Analysis
Source: Comput Struct Biotechnol J. 2026 May 15;35(1):0100. doi: 10.34133/csbj.0100 (PMC13176608; doi:10.34133/csbj.0100)
Supplement: Supplementary 1 — Data S1 to S4 Figs. S1 to S13 [file csbj.0100.f1.zip › TetR_motifs_supplementary_information_rev.docx]

**SUPLEMENTARY DATA**

**Data**

**Data S1**. Positional statistics (median, 25^th^ centile, 75^th^ centile, skewness, kurtosis) and comparisons of pariwise sequence identitiy distributions of sequence sets used for modelling and their clustered at 90, 75 and 50% counterparts. Kolmogorov-Smirnov (KS), Cramer – von Mises(C-vM), Mann – Whitney U (U_MW) tests, Mann-Whitney U effect size and Earth Mover’s distance were calculated with the full count of the used sequence sets against used modelling set.

**Data S2.** The list of functional (highlighted in green) and structural (highlighted in blue) motifs identified in TFRs of known function and structure. For each TFR, the UniProt ID is given. Motif ID is the unique name of the motifs, extracted from the whole set of identified motifs. Start and stop are the positions of the beginning and the end, respectively, of the motif found (matched sequence) in the particular TFR.

**Data S3.** The list of functional and structural motifs listed in Data S2 in .meme format file which can be used for identification of the TFR homolog using MAST from MEME-Suite.

**Data S4.** The list of the analyzed conserved motifs in .meme format file.

**Figures**


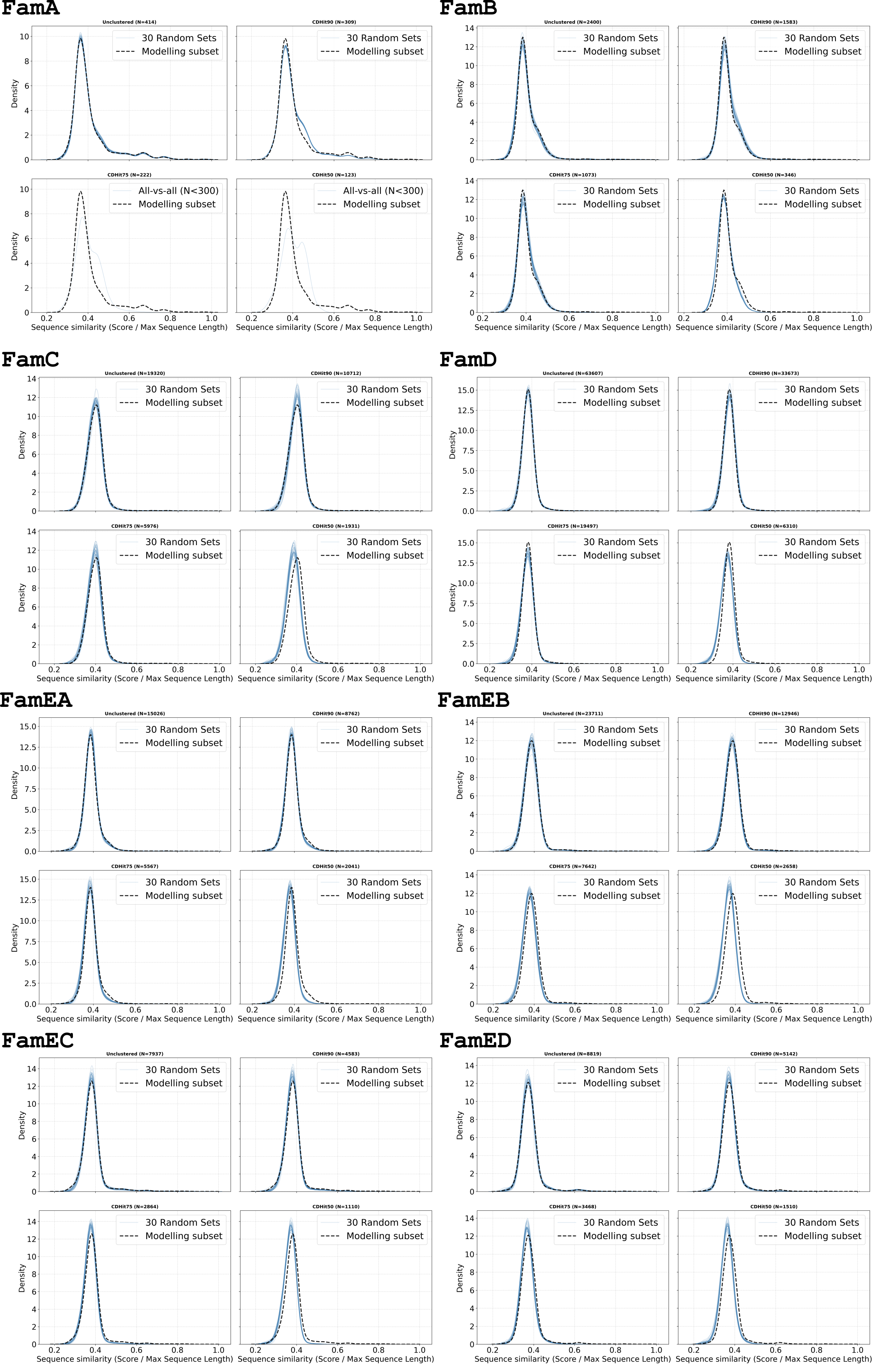


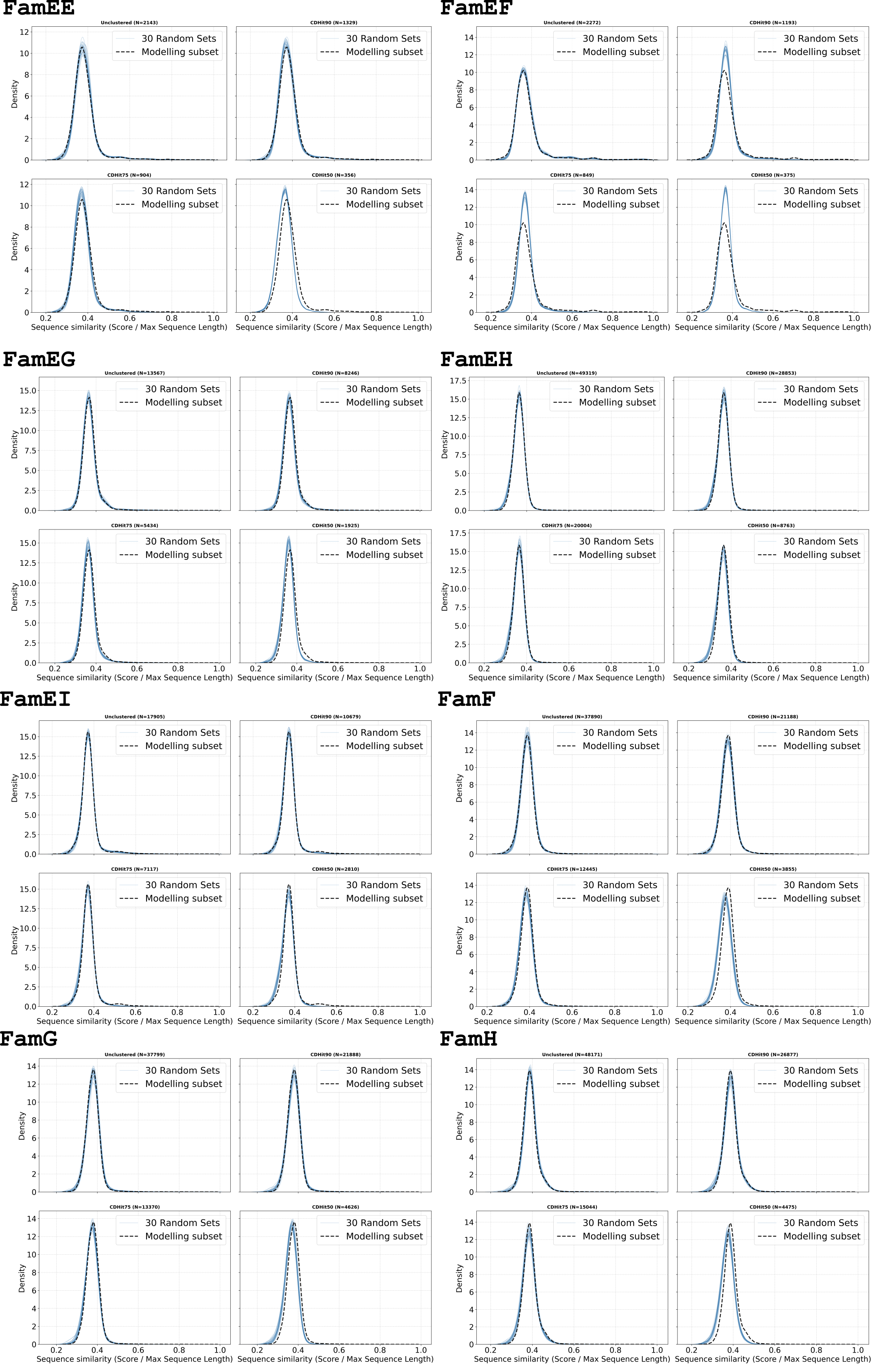


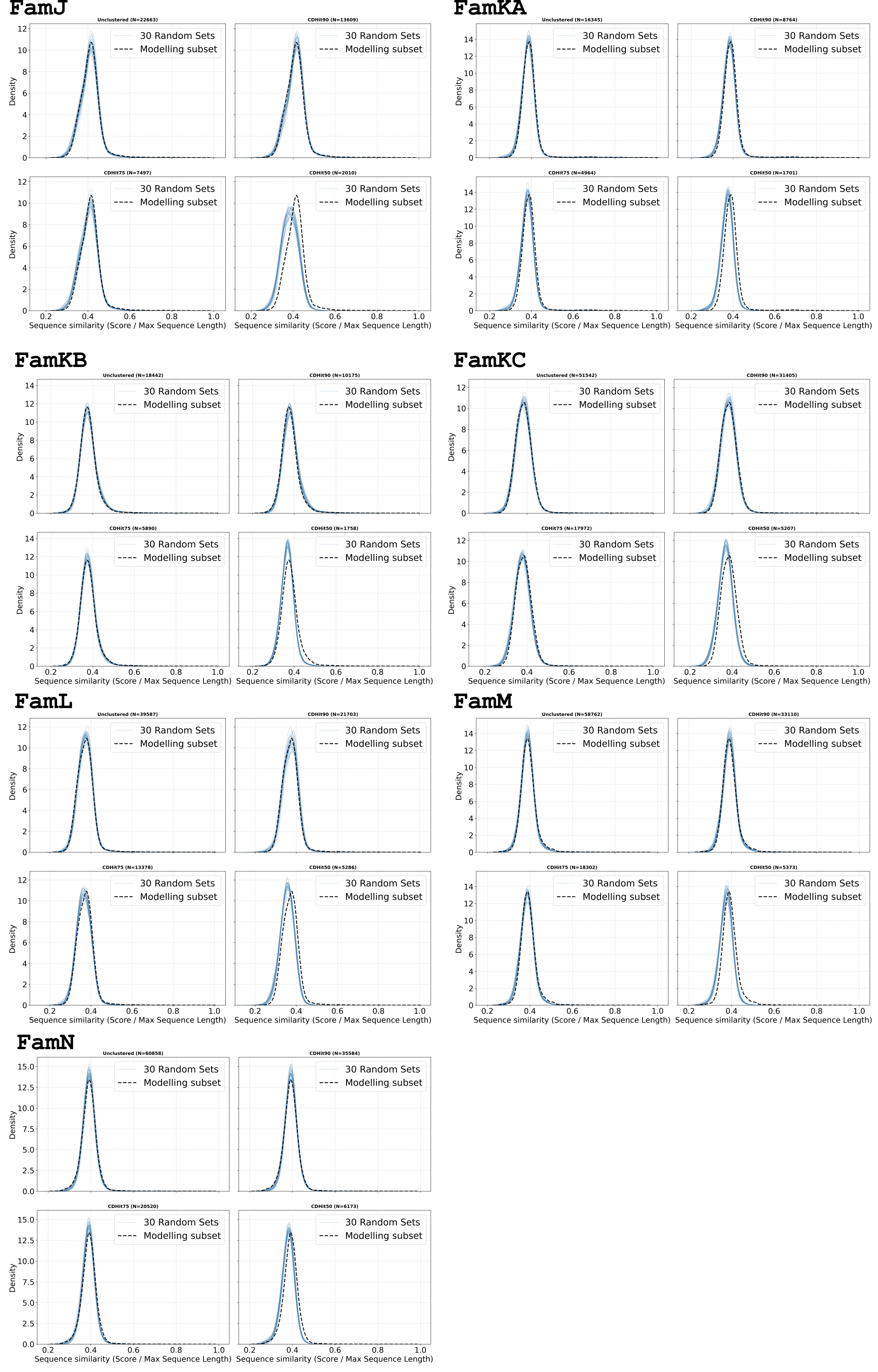


**Fig. S1.** Comparison of pariwise sequence identity distributions in respective TetR subfamilies in unclustered and clustered at 90%, 75% and 50% identity sequence sets. The blue lines indicate the random subsets selected for pairwise sequence identity calculation, the black line is the distribution of the sequence identity calculated for the set of sequences used for modelling. The amount of sequences for each random subset was 300, if the total number of sequences after clustering allowed for selection of that many sequences, otherwise a single distribution of all sequences in respective set is presented.


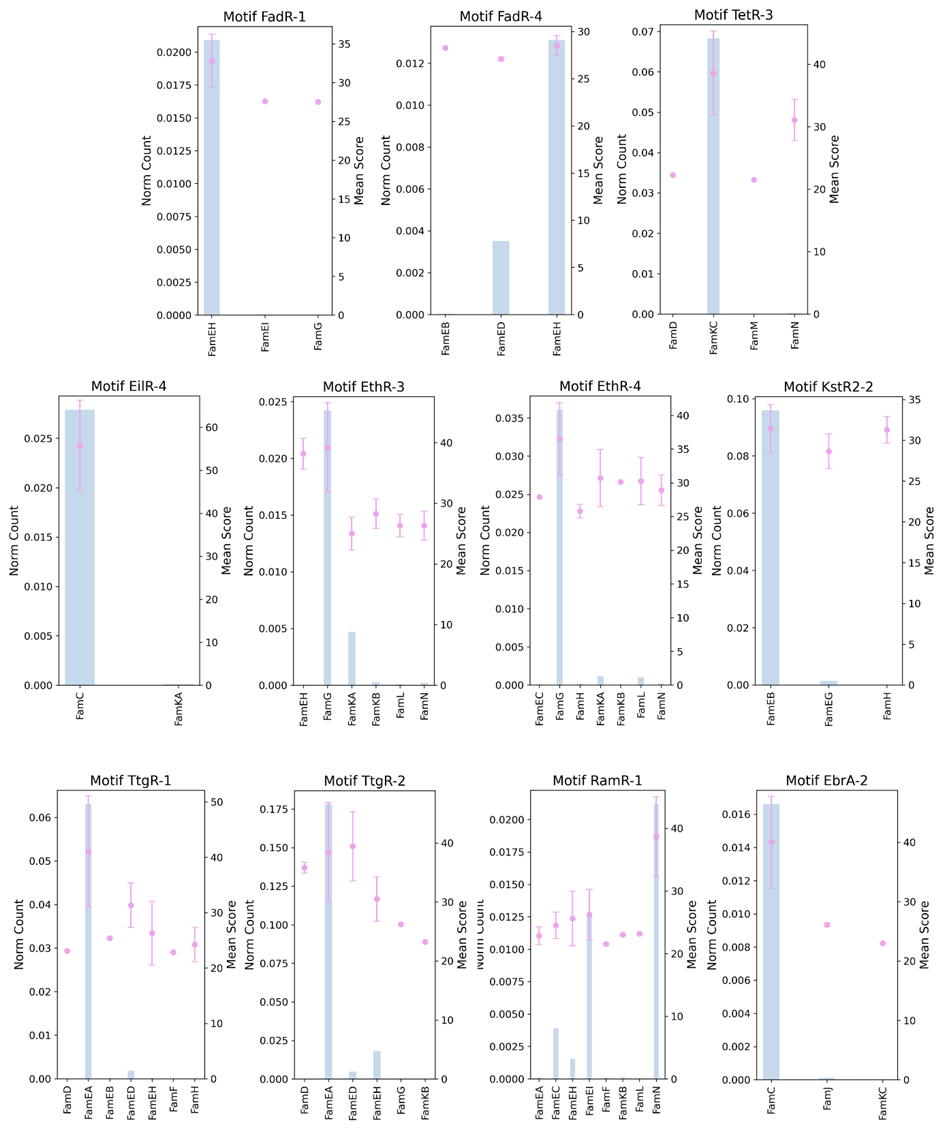


**Fig. S2.** The distribution of the motifs in several subfamilies. Each plot is described with the motif name, the x axis and the bars represent the normalized count of sequences belonging to the subfamily that contain the motif (1 represent all sequences in the subfamily), while the second y axis and the pink whiskers show the mean score calculated for the subfamily.


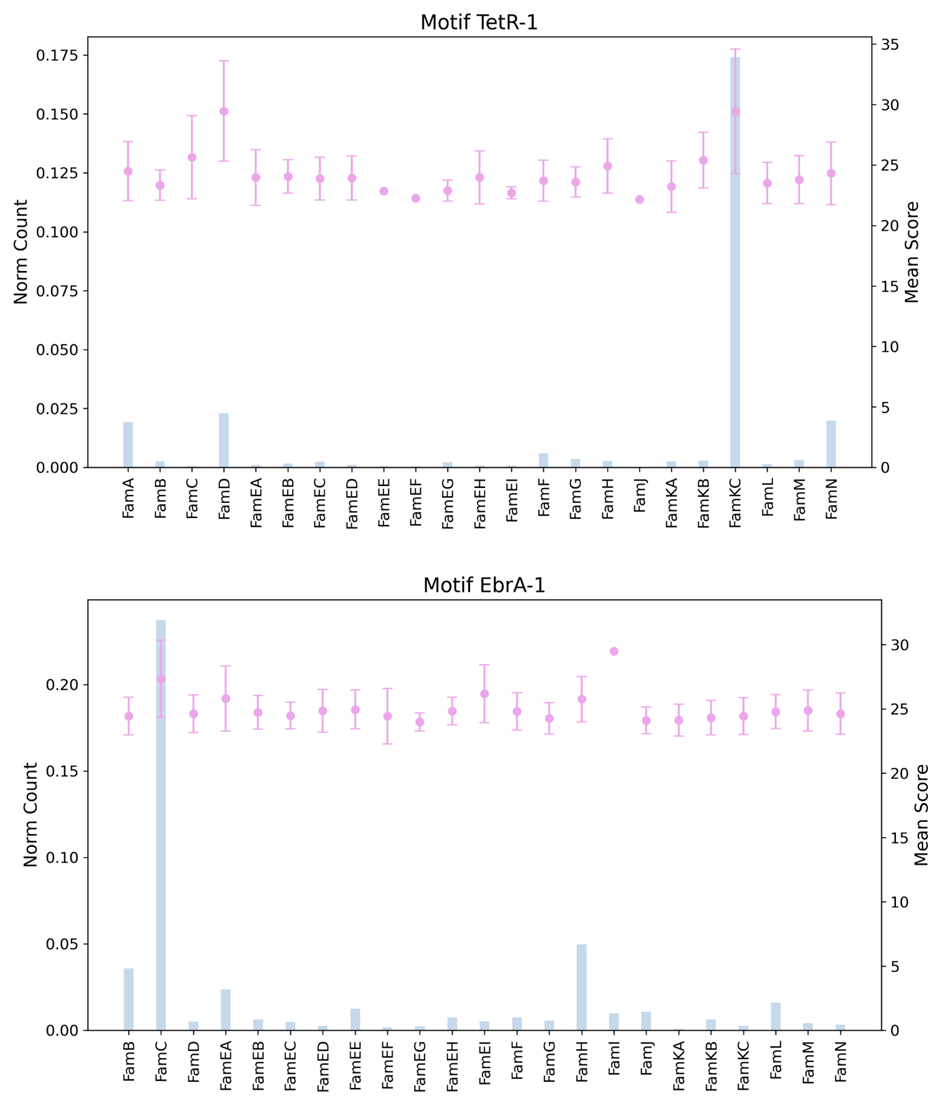


**Fig. S3.** The distribution of the motifs in multiple subfamilies. Each plot is described with the motif name, the x axis and the bars represents the normalized count of sequences belonging to the subfamily that contain the motif (1 represent all sequences in the subfamily), while the secondary y axis and the pink whiskers show the mean score calculated for the subfamily.


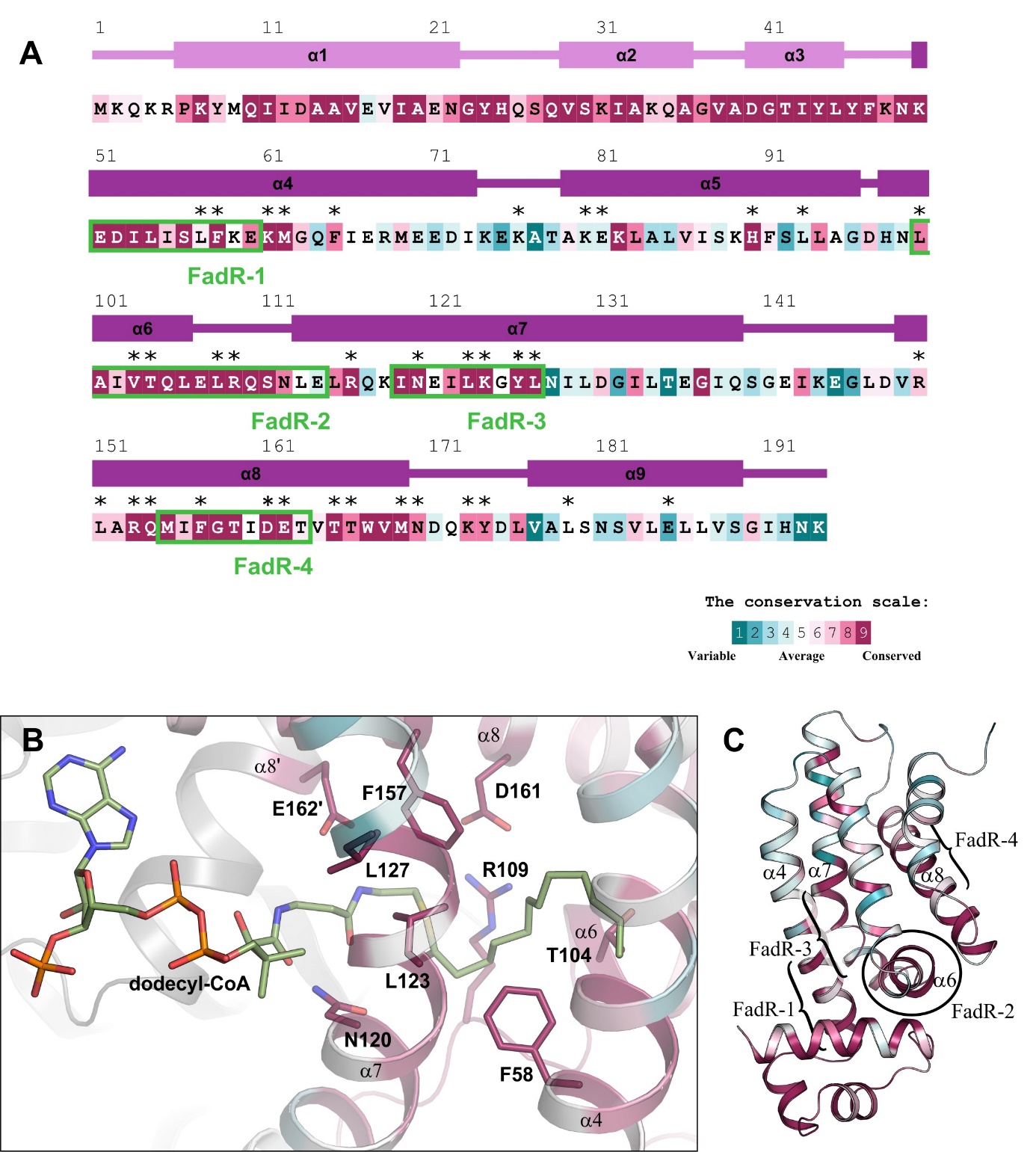


**Fig. S4.** The ConSurf analysis of FadR performed using the crystal structure of Bsu_FadR (PDB ID: 3WHB) and MSA calculated for the sequence set with identified motifs. A) The amino acid sequence of Bsu_FadR colored according to the conservation score calculated by ConSurf. The secondary structure elements are numbered and shown above the sequence. The asterisks indicate the residues interacting with ligands in all known Bsu_FadR structures. B) Ligand-binding site of Bsu_FadR. For clarity, only the residues present in the identified motifs and interacting with the presented ligand are shown as sticks. The helices of chain A are colored according to the ConSurf scale, whereas chain B is presented in light grey with the exception of interacting residues, colored according to conservation score and indicated with prime (’) in its name. C) Cartoon representation of chain A of the Bsu_FadR dimer with the highlighted motifs.


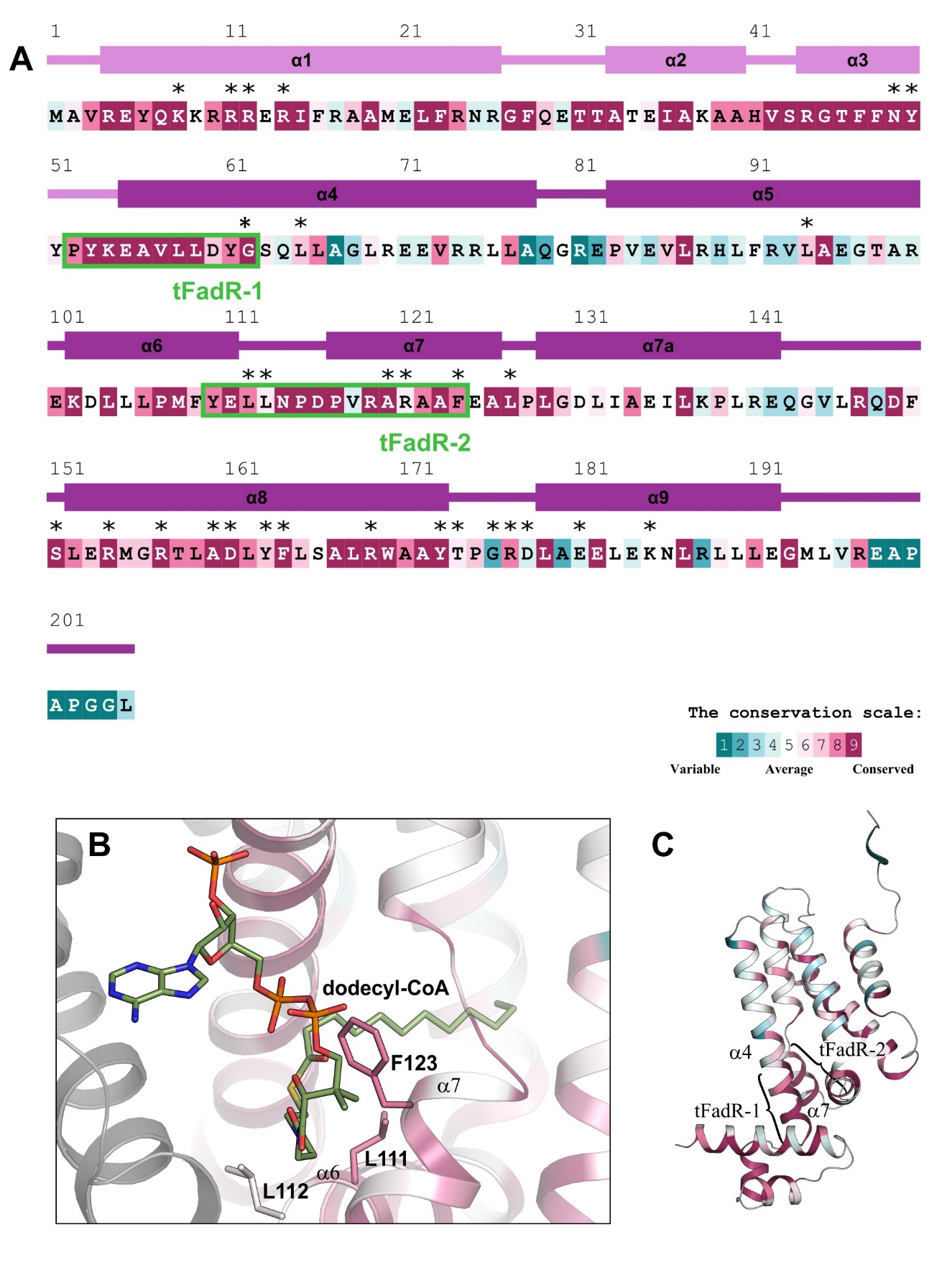


**Fig. S5.** The ConSurf analysis of tFadR performed using the crystal structure of Tth_FadR (PDB ID: 3ANG) and MSA calculated for the sequence set with identified motifs. A) The amino acid sequence of Tth_FadR colored according to the conservation score calculated by ConSurf. The secondary structure elements are numbered and shown above the sequence. The asterisks indicate the residues interacting with ligands in all known Tth_FadR structures. B) Ligand-binding site of Tth_FadR. For clarity, only the residues present in the identified motifs and interacting with the presented ligand are shown as sticks. The helices of chain A are colored according to the ConSurf scale, whereas chain B is presented in light grey. C) Cartoon representation of chain A of the Tth_FadR dimer with the highlighted motifs.


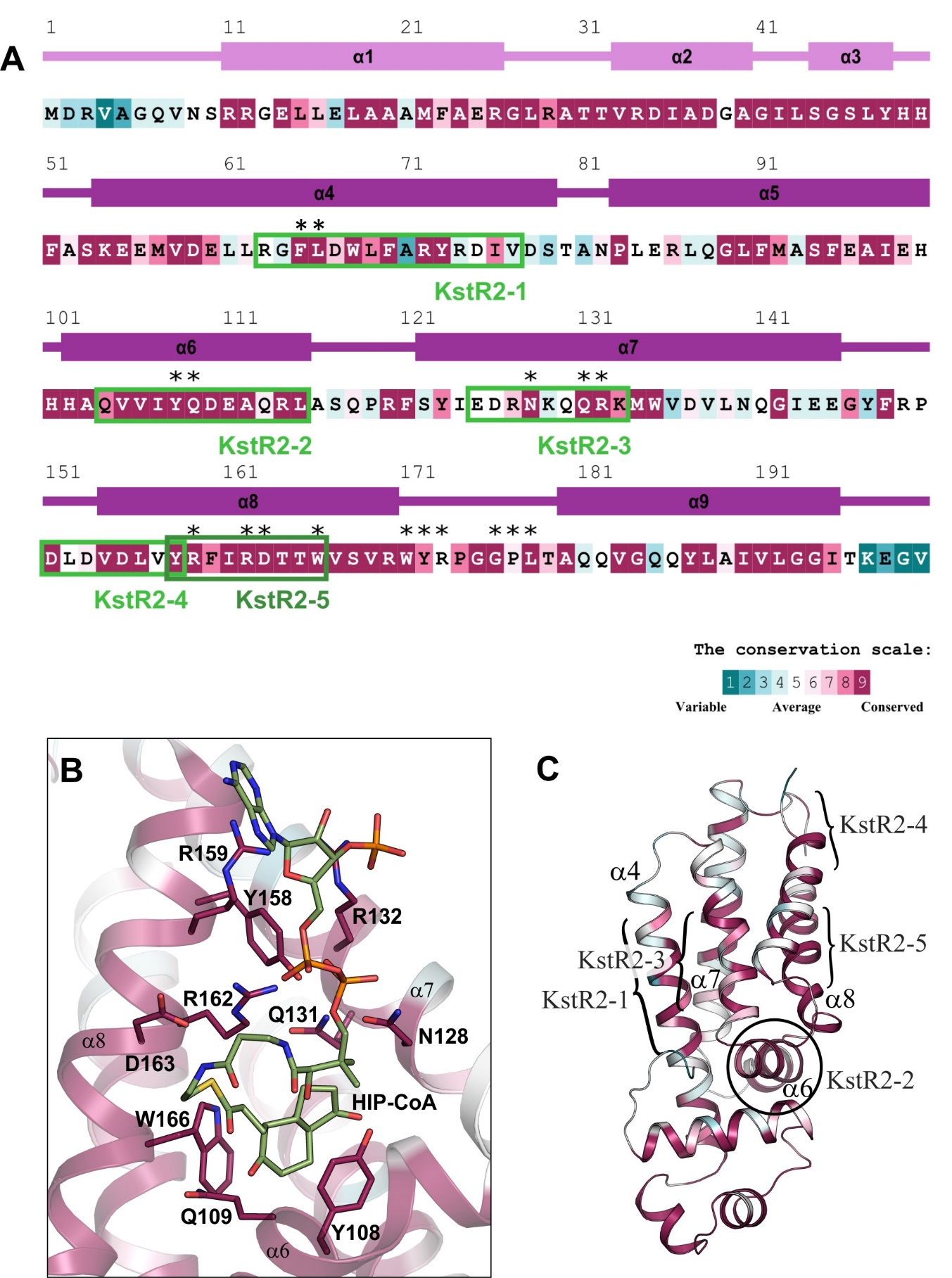


**Fig. S6.** The ConSurf analysis of KstR2 performed using the crystal structure of Mtu_KstR2 (PDB ID: 4W97) and MSA calculated for the sequence set with identified motifs. A) The amino acid sequence of Mtu_KstR2 colored according to the conservation score calculated by ConSurf. The secondary structure elements are numbered and shown above the sequence. The asterisks indicate the residues interacting with ligands in all known Mtu_KstR2 structures. B) Ligand-binding site of Mtu_KstR2. For clarity, only the residues present in the identified motifs and interacting with the presented ligand are shown as sticks. C) Cartoon representation of chain A of the Mtu_KstR2 dimer with the highlighted motifs.


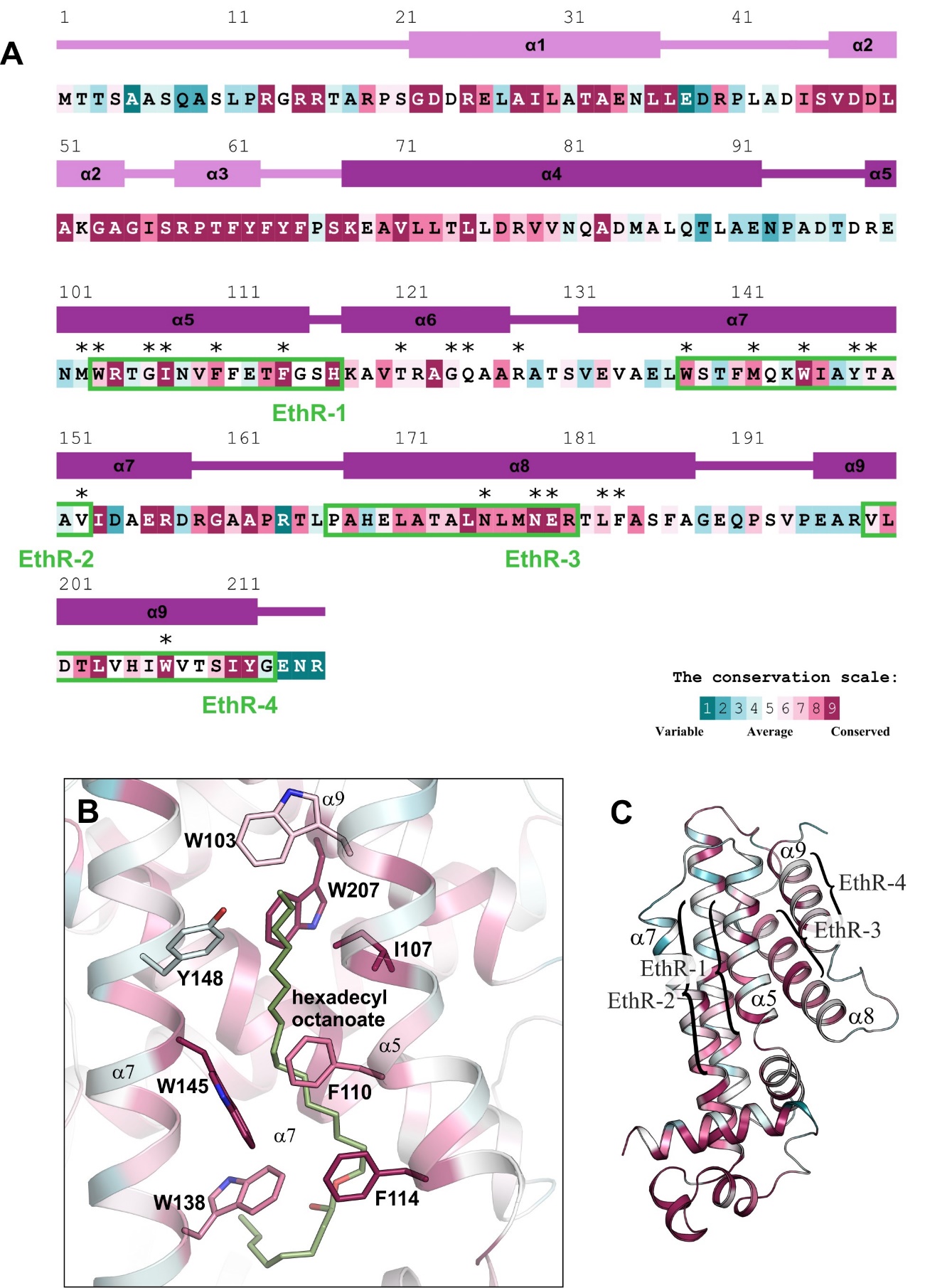


**Fig. S7.** The ConSurf analysis of EthR performed using the crystal structure of Mtu_EthR (PDB ID: 1U9N) and MSA calculated for the sequence set with identified motifs. A) The amino acid sequence of Mtu_EthR colored according to the conservation score calculated by ConSurf. The secondary structure elements are numbered and shown above the sequence. The asterisks indicate the residues interacting with ligands in all known Mtu_EthR structures. B) Ligand-binding site of Mtu_EthR. For clarity, only the residues present in the identified motifs and interacting with the presented ligand are shown as sticks. C) Cartoon representation of chain A of the Mtu_EthR dimer with the highlighted motifs.


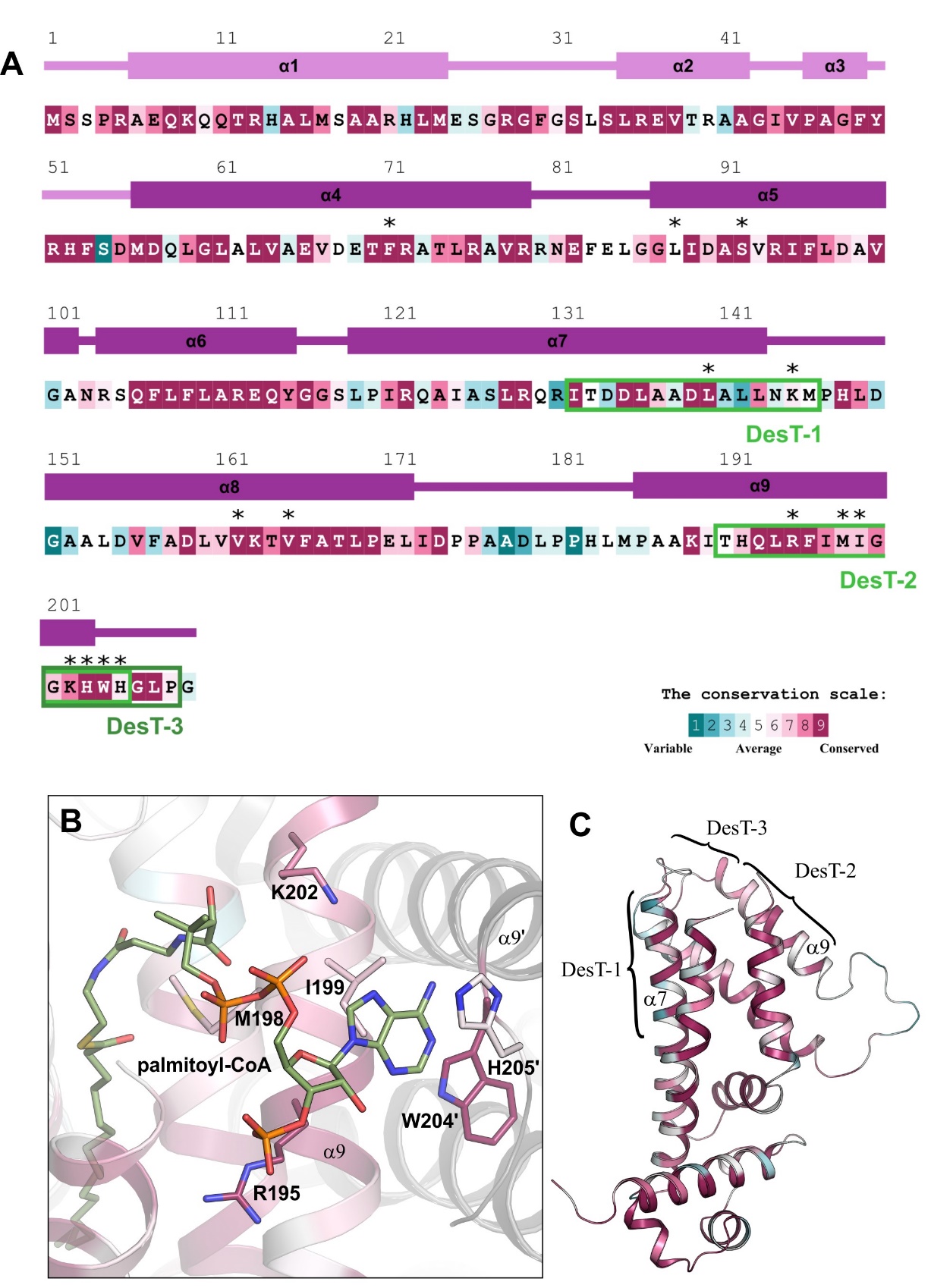


**Fig. S8.** The ConSurf analysis of DesT performed using the crystal structure of Pae_DesT (PDB ID: 3LSJ) and MSA calculated for the sequence set with identified motifs. A) The amino acid sequence of Pae_DesT colored according to the conservation score calculated by ConSurf. The secondary structure elements are numbered and shown above the sequence. The asterisks indicate the residues interacting with ligands in all known Pae_DesT structures. B) Ligand-binding site of Pae_DesT. For clarity, only the residues present in the identified motifs and interacting with the presented ligand are shown as sticks. The helices of chain A are colored according to the ConSurf scale, whereas chain B is presented in light grey with the exception of interacting residues, colored according to conservation score and indicated with prime (’) in its name. C) Cartoon representation of chain A of the Pae_DesT dimer with the highlighted motifs.


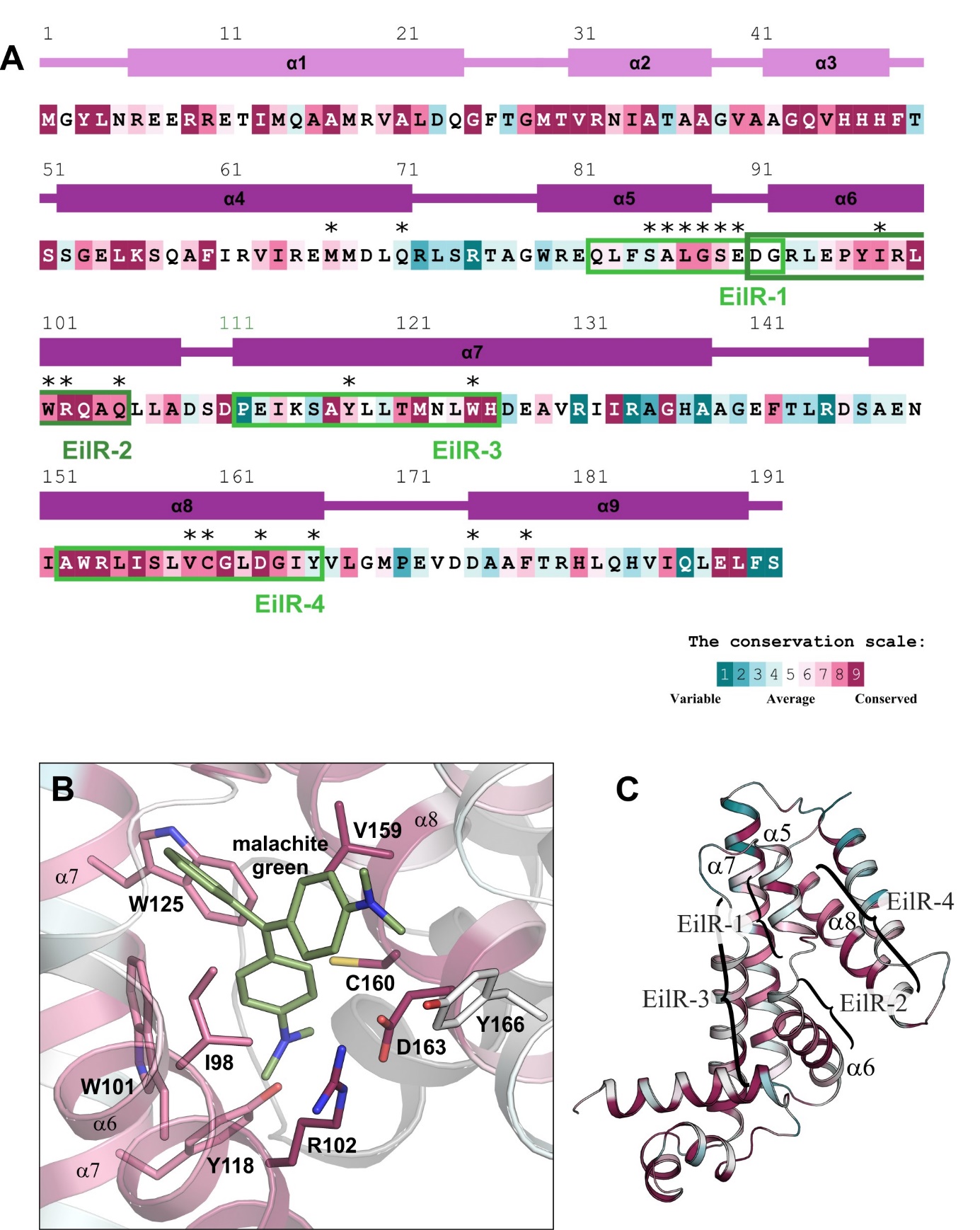


**Fig. S9.** The ConSurf analysis of EilR performed using the crystal structure of Eli_EilR (PDB ID: 5LVG) and MSA calculated for the sequence set with identified motifs. A) The amino acid sequence of Eli_EilR colored according to the conservation score calculated by ConSurf. The secondary structure elements are numbered and shown above the sequence. The asterisks indicate the residues interacting with ligands in all known Eli_EilR structures. B) Ligand-binding site of Eli_EilR. For clarity, only the residues present in the identified motifs and interacting with the presented ligand are shown as sticks. The helices of chain A are colored according to the ConSurf scale, whereas chain B is presented in light grey. C) Cartoon representation of chain A of the Eli_EilR dimer with the highlighted motifs.


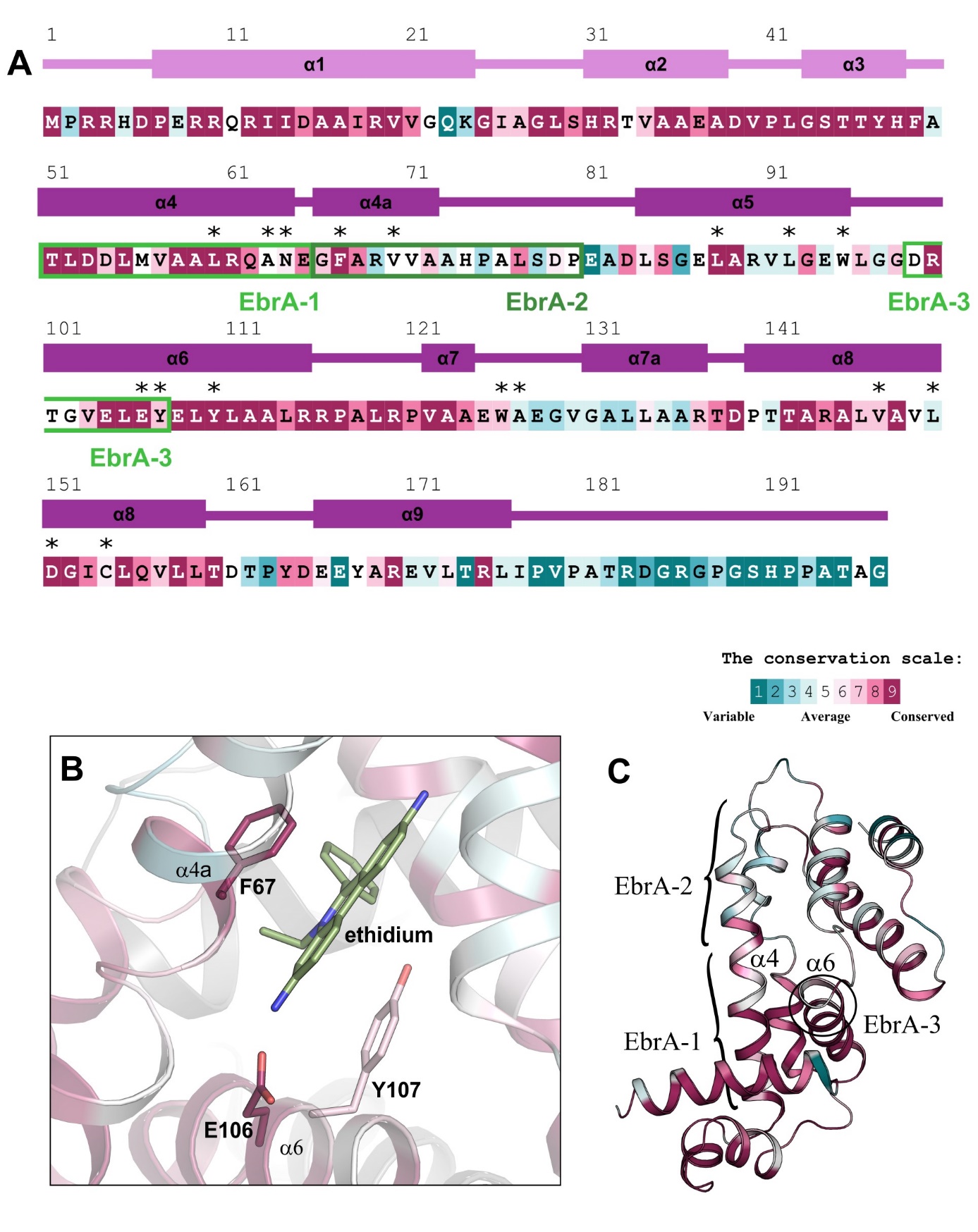


**Fig. S10.** The ConSurf analysis of EbrA performed using the crystal structure of Sli_EbrA (PDB ID: 3HTJ) and MSA calculated for the sequence set with identified motifs. A) The amino acid sequence of Sli_EbrA colored according to the conservation score calculated by ConSurf. The secondary structure elements are numbered and shown above the sequence. The asterisks indicate the residues interacting with ligands in all known Sli_EbrA structures. B) Ligand-binding site of Sli_EbrA. For clarity, only the residues present in the identified motifs and interacting with the presented ligand are shown as sticks. C) Cartoon representation of chain A of the Sli_EbrA dimer with the highlighted motifs.


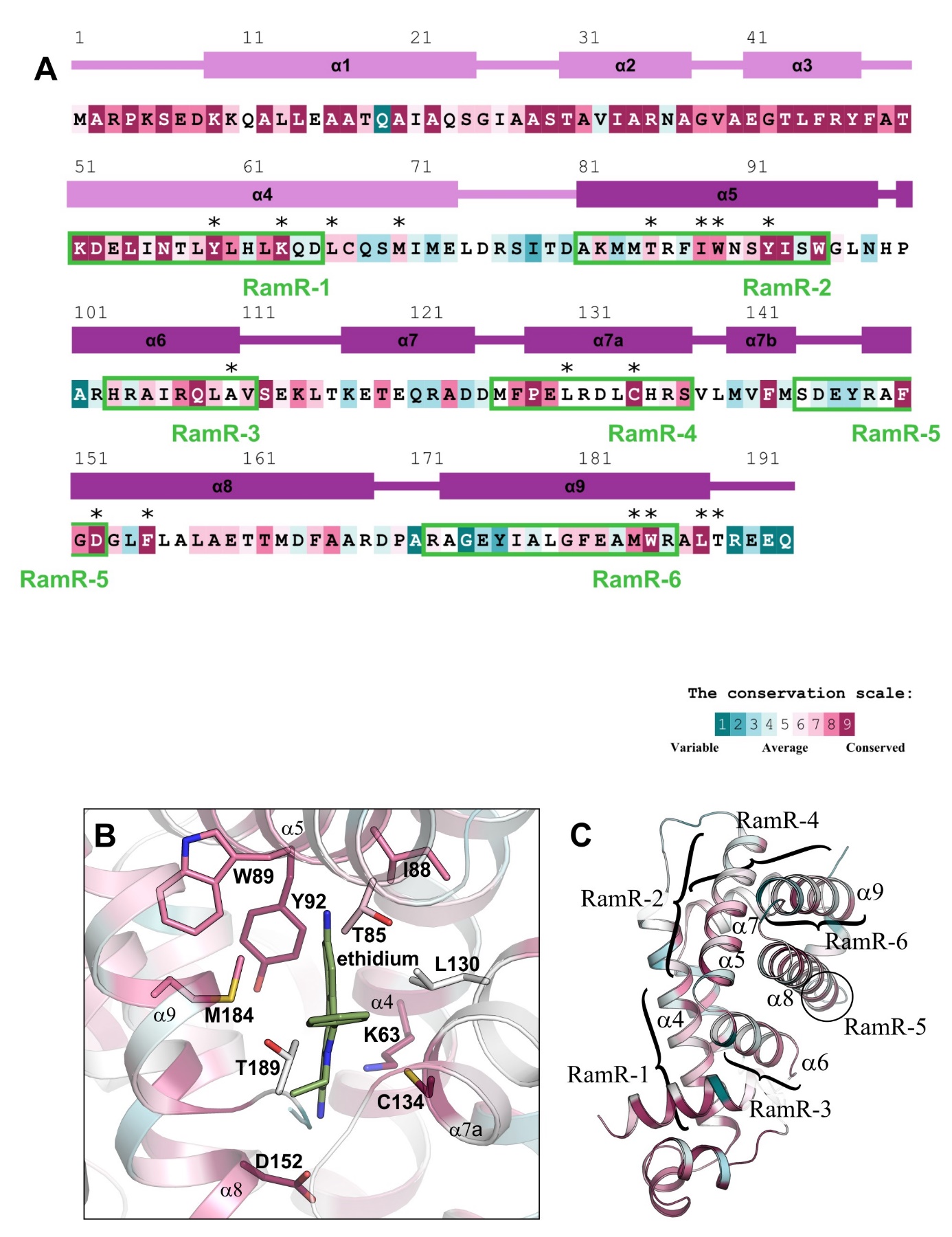


**Fig. S11.** The ConSurf analysis of RamR performed using the crystal structure of Sen_RamR (PDB ID: 6KO7) and MSA calculated for the sequence set with identified motifs. A) The amino acid sequence of Sen_RamR colored according to the conservation score calculated by ConSurf. The secondary structure elements are numbered and shown above the sequence. The asterisks indicate the residues interacting with ligands in all known Sen_RamR structures. B) Ligand-binding site of Sen_RamR. For clarity, only the residues present in the identified motifs and interacting with the presented ligand are shown as sticks. C) Cartoon representation of chain A of the Sen_RamR dimer with the highlighted motifs.


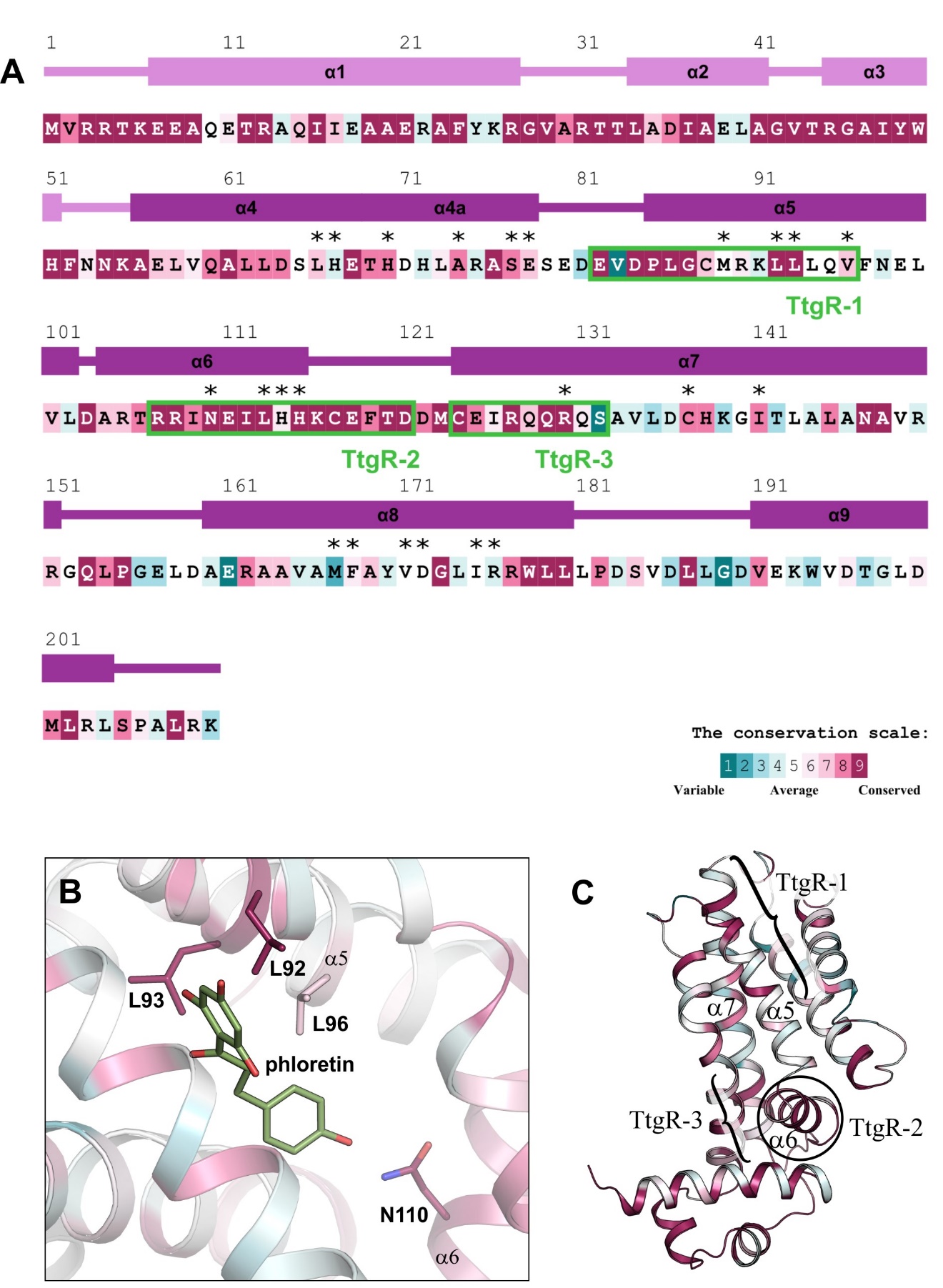


**Fig. S12.** The ConSurf analysis of TtgR performed using the crystal structure of Ppu_TtgR (PDB ID: 2UXI) and MSA calculated for the sequence set with identified motifs. A) The amino acid sequence of Ppu_TtgR colored according to the conservation score calculated by ConSurf. The secondary structure elements are numbered and shown above the sequence. The asterisks indicate the residues interacting with ligands in all known Ppu_TtgR structures. B) Ligand-binding site of Ppu_TtgR. For clarity, only the residues present in the identified motifs and interacting with the presented ligand are shown as sticks. C) Cartoon representation of chain A of the Ppu_TtgR dimer with the highlighted motifs.


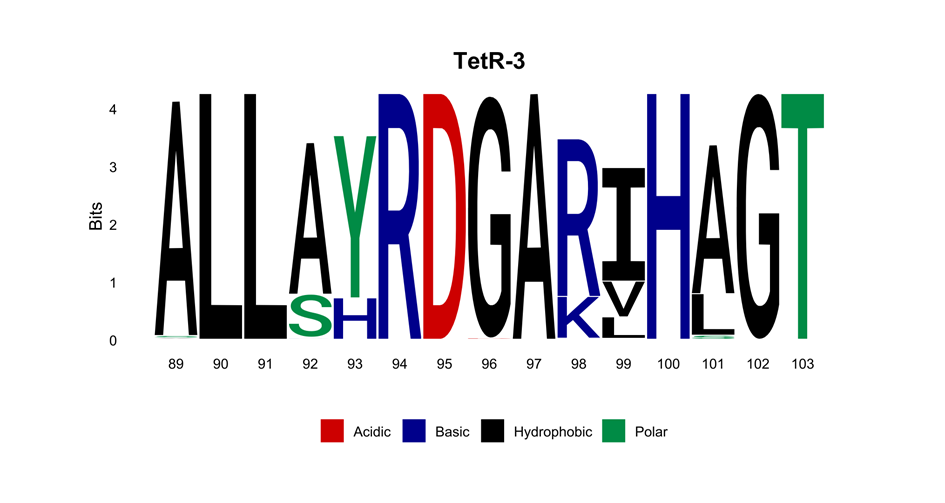
**Fig. S13.** The sequence logo representing the conservation score and compositional variability at each position of the motif TetR-3. The y-axis shows the bit score, where a maximum score of ~4,3 for proteins represents a 100% conservation. The lower the cumulative height of a letter stack reflected by a lower bit score, the bigger the tolerance for substitutions at that position. The relative sizes of amino acids within each stack represent the percentage-wise share of that residue in that position in all aligned sequences, with a dominant amino acid placed on the top of the stack. The x-axis displays the position of a corresponding amino acid in Eco_TetR from which the motif originates. The amino acid residues were colored according to their chemical properties.
